# The use of prescription medications and non-prescription medications during lactation in a prospective Canadian cohort study

**DOI:** 10.1186/s13006-024-00628-x

**Published:** 2024-04-08

**Authors:** Youstina Soliman, Uma Yakandawala, Christine Leong, Emma S. Garlock, Fiona S.L. Brinkman, Geoffrey L. Winsor, Anita L Kozyrskyj, Piushkumar J Mandhane, Stuart E. Turvey, Theo J. Moraes, Padmaja Subbarao, Nathan C. Nickel, Kellie Thiessen, Meghan B Azad, Lauren E Kelly

**Affiliations:** 1 Department of Pediatrics and Child Health, University of Manitoba, Winnipeg, MB Canada; 2 George and Fay Yee Centre for Healthcare Innovation, Winnipeg, MB Canada; 3 College of Pharmacy, Rady Faculty of Health Science, University of Manitoba, Winnipeg, MB Canada; 4 Department of Molecular Biology and Biochemistry, Simon Fraser University, Burnaby, BC Canada; 5 Department of Pediatrics, Faculty of Medicine & Dentistry, University of Alberta, Edmonton, AB Canada; 6 Department of Pediatrics, University of British Columbia, Vancouver, Canada; 7 Department of Pediatrics, Hospital for Sick Children, University of Toronto, Toronto, Canada; 8 Department of Community Health Sciences, University of Manitoba, Winnipeg, MB Canada; 9 Manitoba Interdisciplinary Lactation Centre (MILC), Winnipeg, MB Canada; 10 College of Nursing, Rady Faculty of Health Sciences, University of Manitoba, Winnipeg, MB Canada; 11 Manitoba Interdisciplinary Lactation Centre (MILC), Children’s Hospital Research Institute of Manitoba, Winnipeg, MB Canada; 12 417-753 McDermot Ave, R3E 0T6 Winnipeg, MB Canada

**Keywords:** Cannabinoids, Chronic headache, Clinical trials, Adolescent, Pain

## Abstract

**Background:**

A lack of safety data on postpartum medication use presents a potential barrier to breastfeeding and may result in infant exposure to medications in breastmilk. The type and extent of medication use by lactating women requires investigation.

**Methods:**

Data were collected from the CHILD Cohort Study which enrolled pregnant women across Canada between 2008 and 2012. Participants completed questionnaires regarding medications and non-prescription medications used and breastfeeding status at 3, 6 and 12 months postpartum. Medications, along with self-reported reasons for medication use, were categorized by ontologies [hierarchical controlled vocabulary] as part of a large-scale curation effort to enable more robust investigations of reasons for medication use.

**Results:**

A total of 3542 mother-infant dyads were recruited to the CHILD study. Breastfeeding rates were 87.4%, 75.3%, 45.5% at 3, 6 and 12 months respectively. About 40% of women who were breastfeeding at 3 months used at least one prescription medication during the first three months postpartum; this proportion decreased over time to 29.5% % at 6 months and 32.8% at 12 months. The most commonly used prescription medication by breastfeeding women was domperidone at 3 months (9.0%, *n* = 229/2540) and 6 months (5.6%, *n* = 109/1948), and norethisterone at 12 months (4.1%, *n* = 48/1180). The vast majority of domperidone use by breastfeeding women (97.3%) was for lactation purposes which is off-label (signifying unapproved use of an approved medication). Non-prescription medications were more often used among breastfeeding than non-breastfeeding women (67.6% versus 48.9% at 3 months, *p* < 0.0001), The most commonly used non-prescription medications were multivitamins and Vitamin D at 3, 6 and 12 months postpartum.

**Conclusions:**

In Canada, medication use is common postpartum; 40% of breastfeeding women use prescription medications in the first 3 months postpartum. A diverse range of medications were used, with many women taking more than one prescription and non-prescription medicines. The most commonly used prescription medication by breastfeeding women were domperidone for off-label lactation support, signalling a need for more data on the efficacy of domperidone for this indication. This data should inform research priorities and communication strategies developed to optimize care during lactation.

**Supplementary Information:**

The online version contains supplementary material available at 10.1186/s13006-024-00628-x.

## Background

Breastfeeding has various important maternal and child health benefits which includes facilitating maternal-infant bonding and delivering necessary nutrients and antibodies for the infant’s developing immune system [1]. Exclusive breastfeeding for the first six months and then continuing breastfeeding up to two years or more after introducing solid foods is recommended by many organizations including World Health Organization (WHO) and Health Canada [1, 2]. In Canada, breastfeeding initiation rates in hospital are high at 89% (reported in 2011/12), however, continued rates of breastfeeding post-discharge are much lower, as only 26% of mothers reported breastfeeding exclusively for six months or more [3]. Support and encouragement for mothers who wish to breastfeed is an important public health initiative which includes a thorough investigation of barriers to sustained breastfeeding.

Medication use, without adequate safety data, is a potential barrier to breastfeeding due to risks of infant exposure through breastmilk [4]. Mothers may have acute or chronic health conditions that require the use of medications in the postpartum period. Prescription medications, non-prescription medications (NPM) such as ibuprofen or allergy medicines, and supplements may be necessary for their clinical care and well-being. Medication transfer and safety during lactation are rarely studied during drug development [4, 5]. Mothers requiring medications and their health care providers may be ill-informed about the risks of nursing while taking medications and supplements [4]. This can result in an absolute risk avoidance approach to either stop breastfeeding or discontinue taking medications to avoid possible (unknown) adverse effects on their child [4]. It is critical for health professionals to assess the risk and benefits for both mother and child, including the risks of not breastfeeding, when advising on postpartum medication decisions for women who wish to breastfeed. Most medications are considered relatively safe for infants that are breastfed [5]. There are a few medications that might be relatively contraindicated during breastfeeding which include lithium, oral retinoids, amiodarone, gold salts and anticancer medications [6]. For most medications further knowledge is required to understand the effects during breastfeeding. It is recommended that health care providers review medications on a case-by-case basis for suitability and advice regarding risk management. A study by Hale et al., reported that common medications used in the postpartum period include analgesics, antihypertensives, sedatives, antidepressants, antipsychotics, antiepileptics, antibiotics, and steroids; however, breastfeeding patterns and rates were not reported [5].

The primary aim of the present study is to identify the most commonly used prescription medications and NPM among breastfeeding mothers in a Canadian prospective cohort study. Our secondary aims were to characterize patterns of medication use based on therapeutic categories among breastfeeding women and non-breastfeeding women, describe indications of prescription medication use, and explore differences in medication use by study site.

## Methods

### Study population

Data were collected from the Canadian CHILD Cohort Study [7]. In the CHILD study, pregnant women in their second and third trimester were recruited between 2008 and 2012 at 4 sites across Canada - Vancouver, Edmonton, Manitoba (Winnipeg, Morden, Winkler) and Toronto [7]. Women were eligible to participate if they gave birth to a singleton infant at > 35 weeks gestational age. Infants who were born premature (≤ 35 weeks), born with respiratory distress syndrome, complications or fetal abnormalities or born through an in vitro fertilization were excluded [7]. A total of 3542 mother-infant dyads were originally recruited to the CHILD study [7]. The CHILD study was approved by the Human Research Ethics Boards at McMaster University and at the Universities of Alberta, British Columbia, and Manitoba, and the Hospital for Sick Children in Toronto [8]. Our subsequent analysis was approved by the Health Research Ethics Bord of the University of Manitoba.

### Medication classification and breastfeeding status

Each participant was asked to complete a questionnaire at 3, 6 and 12 months postpartum identifying any prescription medications or NPMs including vitamins and supplements used during the first 3 months postpartum, 3 to 6 months postpartum and 6 to 12 months postpartum, specifying the medication/product type. Participants were also able to self-report their reason for using a specific prescription medication, but this was not required. There was no option to report indication for NPM. The reported medications were classified as prescription medications or NPM, based on the individual drug monographs as per Drug Product Database online query from Health Canada which classifies prescription medications or NPMs according to Food and Drug Regulations, and the Controlled Drugs and Substances Act [9].. Prescription medications and NPM were then further classified based on their therapeutic class based on the drug database, Lexicomp [10]. Information about breastfeeding status was obtained at 3, 6 and 12 months postpartum from self-reported questionnaires. Breastfeeding was defined using WHO definition as “breast milk, including milk that was expressed” and therefore includes pumped milk [11]. Mothers were classified into the breastfeeding versus the non-breastfeeding group at each time point postpartum.

### Large scale data curation with ontologies, and statistical analyses

**Fig. 1:**
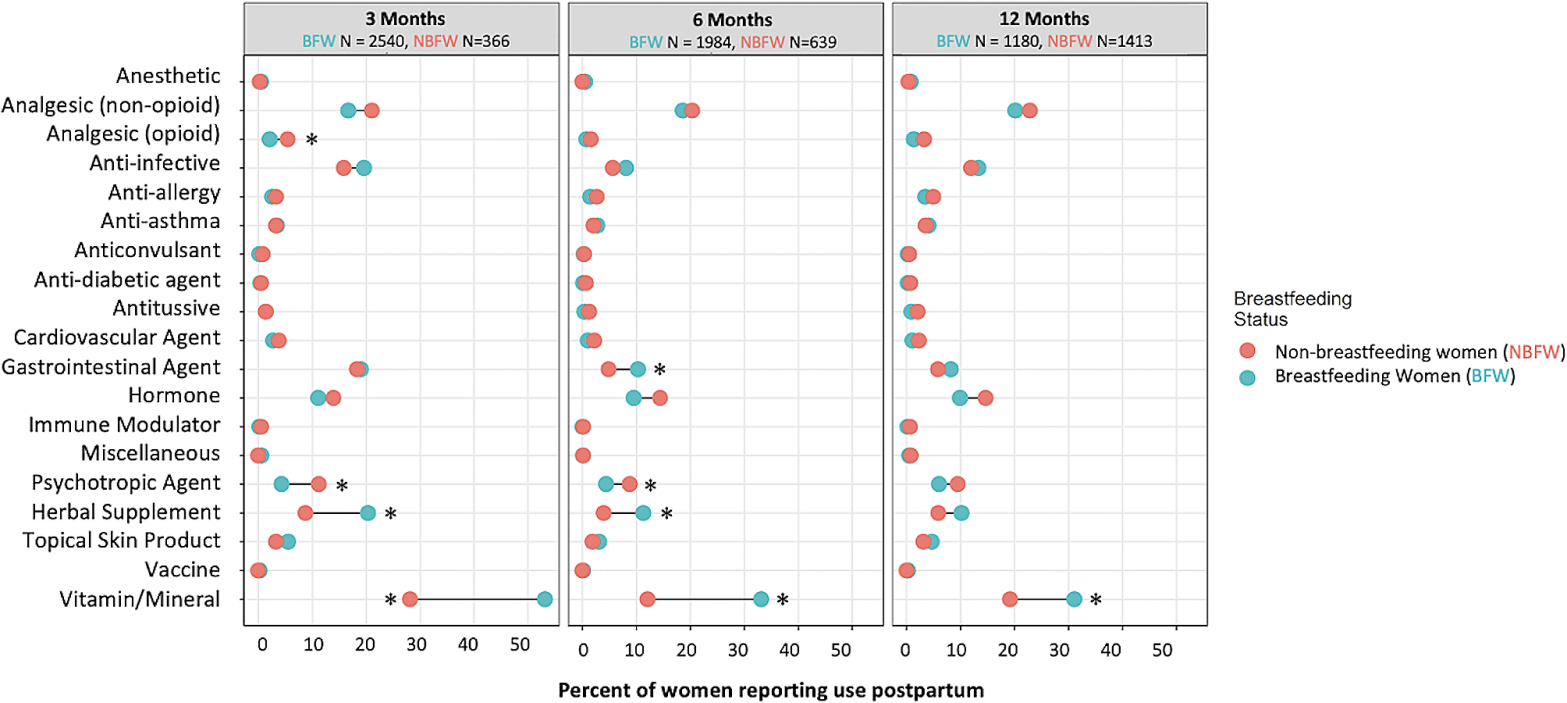
Prescription medication and non-prescription medications usage at least once by breastfeeding and non-breastfeeding women at 0–3, 3–6 and 6–12 months in the CHILD Cohort Study. * denotes statistical significance

Medication brand names were corrected for spelling errors and the generic names of the medications were then identified. When the brand name was not specific, a broad ontology term was provided instead of the generic name. The most common prescription medications and NPM used by breastfeeding women (BFW) for each time point were described by study site to identify signals of regional variability. To analyze reasons for medication use, free text entries were corrected for spelling errors and then standardized using ontology terms from Human Disease Ontology, Human Phenotype Ontology, National Cancer Information Thesaurus, Adverse Event Ontology, and Symptom Ontology. There was an initial automated analysis through the Disease Ontology (DOID), Human Phenotype Ontology (HPO), Symptom Ontology (SYMP), Ontology of Adverse Events (OAE) and the National Cancer Institute Thesaurus (NCIT) using a Ontoma python wrapper to match the free text to the above defined ontology [12–16]. This was followed by extensive curation of the 4426 unique reasons for medication use. Reasons for medication use summaries were then based on the ontology term instead of the free text entry. Fisher’s Exact test was used to analyze categorical differences in the usage patterns of prescription medication and other health care products for BFW and non-breastfeeding women (NBFW) at each time point. A two-tailed P was calculated based on this test. This analysis was conducted to compare number of medications used as well as differences in usage patterns of medications based on the therapeutic category and a FDR correction was used for statistical analysis with level of significance of *p* < 0.05 (Fig. 1).

## Results

### Study population and breastfeeding rates

A total of 3542 mother-infant dyads were originally recruited to the CHILD study. A total of 2906, 2587 and 2593 participants indicated their breastfeeding status at 3, 6 and 12 months respectively and were included in this analysis, with 2060 providing breastfeeding data at all three timepoints. Characteristics of BFW and NBFW are described in Table 1. A total of 87.4% (*n* = 2540/2906), 75.3% (*n* = 1948/2587), 45.5% (*n* = 1180/2593) of participants were breastfeeding at 3, 6 and 12 months respectively. Breastfeeding women were found to be older and more educated than non-breastfeeding women. Women who were breastfeeding at 3 months and 6 months postpartum had higher annual household income than their non-breastfeeding counterparts however this trend did not persist at 12 months postpartum. There was a higher proportion of women who breastfeeding than not breastfeeding in Vancouver at 3, 6 and 12 months postpartum however lower breastfeeding proportions were found in Edmonton and Manitoba at these time points. Toronto had equivalent proportion of breastfeeding versus non-breastfeeding women at 3 and 6 months postpartum however lower breastfeeding rates at 12 months postpartum.

### Patterns of medication usage in BFW and NBFW

The number of prescription medications used by BFW and NBFW was not significantly different at 3, 6 and 12 months postpartum. At 3 months postpartum, 41.9% of BFW and 45.9% of NBFW reported using at least one prescription medication during the first 3 months postpartum. At 6 months postpartum, 29.5% of BFW and 30.7% of NBFW reported using at least one prescription medication during the 3–6 months postpartum period. At 12 months postpartum, 32.8% of BFW and 37.6% of NBFW reported using at least one prescription medication during the 6–12 months postpartum period. Domperidone was among the top 3 most commonly used prescription medication by BFW at 3, 6 and 12 months. Given that domperidone was largely used for lactation purposes, it was initially thought that it could have contributed to the large number of breastfeeding women taking prescription medications in this study as shown in Fig. 1. However, even when domperidone was excluded from the data, there were no differences in the number of prescriptions that that were reported by BFW and NBFW.

In contrast, when comparing usage of NPM, there were significantly more BFW who reported using at least one NPM than NBFW. At 3 months postpartum, 67.6% of BFW and 48.9% of NBFW used at least one NPM (*p* < 0.0001). The median (range) number of NPM used at 3 months is 1 (0–12) and 0 (0–8) for BFW and NBFW women respectively. At 6 months postpartum, 47.8% of BFW and 30.5% of NBFW used at least one NPM (*p* < 0.0001); 0 (0–14) vs. 0 (0–10). At 12 months postpartum, 46.9% of BFW and 39.6% of NBFW used at least one NPM (*p* < 0.001); 0 (0–12) vs. 0 (0–10).

### Most commonly used medications and their indications

**Fig. 2:**
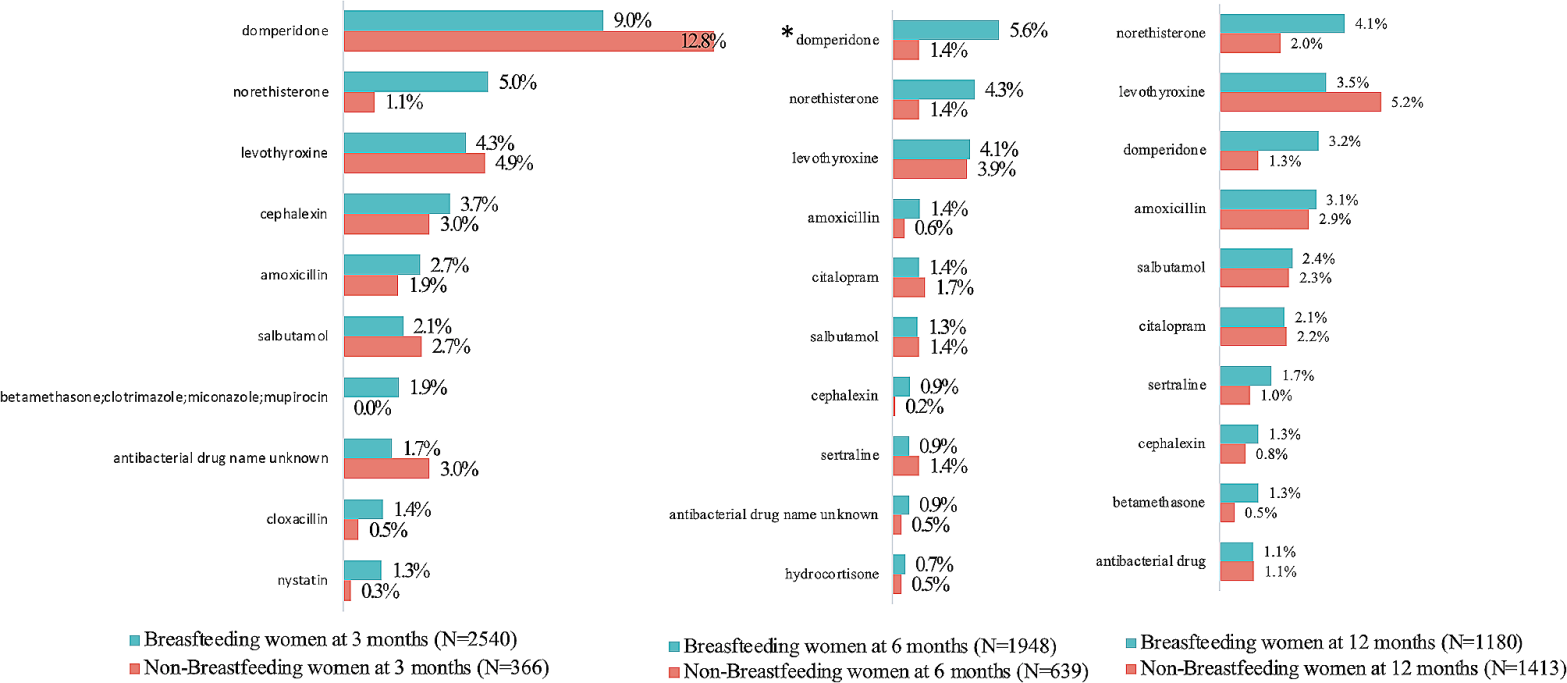
The 10 most common prescription medications used by breastfeeding mothers during the first 3 months, 3 to 6 months, and 6 to 12 months in the CHILD Cohort Study. Usage compared to non-breastfeeding mothers. * denotes statistical significance

The ten most commonly used prescription medication by BFW at 3, 6 and 12 months are shown in Fig. 2. The five most commonly used prescription medication by BFW at 3 months was domperidone (9.0%, *n* = 229/2540), norethisterone (5.0%, *n* = 128/2540), levothyroxine (4.3%, *n* = 108/2540), cephalexin (3.7%, *n* = 94/2540), amoxicillin (2.7%, *n* = 68/2540).

Tables 2, 3 and 4 contain the ontologized self-reported reasons for use of these top ten medications for each time point in the same respective order. The self-reported reasons for medication use of domperidone included off-label use to manage lactation (signifying unapproved use of an approved medication). Breastfeeding mothers reported using the antimicrobials cephalexin (3.7%, *n* = 94/2540), amoxicillin (2.7%, *n* = 68/2540), cloxacillin (1.4%, *n* = 35/2540) and nystatin (1.3%, *n* = 24/2540) to combat mastitis, unspecific infections, and other breast abnormalities. The complete lists of prescription and NPMs and their usage pattern by BFW and NBFW are shown in Additional file 2 and 3.

The five most common NPMs used by BFW at 3 months was multivitamin (48.3%, *n* = 1226/2540), vitamin D (16.5%, *n* = 419/2540), acetaminophen (10.4%, *n* = 264/2540), calcium (8.4%, *n* = 213/2540), omega 3 (8.0%, *n* = 203/2540). The five most common non-prescription medications used by BFW at 6 months was multivitamin (28.9%, *n* = 563/1948), acetaminophen (11.8%, *n* = 230/1948), vitamin D (11.5%, *n* = 224/1948), ibuprofen (7.6%, *n* = 148/1948), calcium (5.3%, *n* = 103/1948). The five most common non-prescription medications used by BFW at 12 months was multivitamin (25.9%, *n* = 306/1180), vitamin D (12.5%, *n* = 148/1180), acetaminophen (11.6%, *n* = 137/1180), ibuprofen (9.4%, *n* = 111/1180), omega 3 (5.4%, *n* = 64/1180). Indications were not reported for NPMs.

### Medication use by site

The most common prescription medications and NPM for each time point were also compared by study site for BFW to evaluate regional patterns as seen in Additional file 2 and 3. At 3 months (but not 6 or 12 months), there was a significant difference for the usage of domperidone in BFW between participant sites, with higher usage in Vancouver compared to the other sites (Edmonton 8.8%, 46/521; Toronto 7.5%, 46/613; Vancouver 13%, 86/661; Manitoba 6.8%, 51/745; <0.001). For NPM at 3 months postpartum, there was a significance difference for the usage of multivitamins for BFW, with more than 2-fold higher usage in Vancouver compared to Manitoba (Edmonton 45.3%, 236/521; Toronto 54.5%, 334/613; Vancouver 69.9%, 462/661; Manitoba 26.3%, 196/745; <0.0001). These differences persisted at 6 months but were no longer evident at 12 months. At 12 months, there was a significant difference observed for norethisterone usage in BFW, with higher usage in Edmonton and Manitoba compared to Toronto and Vancouver (Edmonton 8.1%, 17/211; Toronto 2.4%, 5/209; Vancouver 1.3%, 5/400; Manitoba 5.8%, 21/360; <0.0001).

### Pattern of medication use based on therapeutic categories in BFW and NBFW

Prescription medications and other medications used by women postpartum are shown in Fig. 1, grouped into therapeutic categories. There were several significant differences in therapeutic class usage frequency between BFW and NBFW. At 3 months postpartum, BFW were more likely to use supplement/herbal remedies (20.4% versus 8.7%, < 0.0001) and vitamins/minerals (53.1% versus 28.1%, < 0.0001), whereas NBFW were more likely to use opioid analgesics (2.1% versus 5.5%, 0.003) and psychotropic agents (4.3% versus 11.2%, < 0.0001). At 6 months, BFW remained more likely to use supplement/herbal remedies (11.3% versus 3.9%, < 0.0001) and vitamins/minerals (33.2% versus 12.1%, < 0.0001) and were also more likely to use gastrointestinal agents (10.3% versus 4.9%, 0.0001); NBFW remained more likely to use psychotropic agents (4.4% versus 8.8%, < 0.0001). At 12 months, BFW remained more likely to use vitamins/minerals (31.1% versus 19.2%, < 0.0001) but no other differences were observed. All other therapeutic categories, such as non-opioid analgesics, anti-infectives, cardiovascular agents, etc., had no observed significant differences in the breastfeeding versus the non-breastfeeding groups.

## Discussion

Given the importance and relative knowledge gap on patterns and reasons for use of medication and supplement use during lactation, our team performed an analysis investigating the most commonly used medications and health supplements during lactation, alongside a novel ontologiza (assigned into a category), across a diverse Canadian cohort. Our study reports that about 40% of BFW were using at least one prescription medication in the first 3 months postpartum (41.9%), and that usage decreased over time (29.5% at 6 months and 32.8% at 12 months). For comparison, a study conducted in Brazil looking at medication use in the first 12 months postpartum or until weaning, also found 52.4% of women used prescription medications while breastfeeding [17].

At 3 and 6 months postpartum, the most commonly used prescription medication was domperidone, which was used more commonly in NBFW than BFW in the first 3 months postpartum in our cohort. Domperidone is widely used as an antiemetic and motility agent that has been used off-label to improve lactation during breastfeeding [18]. Recent reports highlight safety concerns of domperidone including potential withdrawal concerns and heart problems [19, 20]. Analysis of self-reported reasons for use among participants confirmed that domperidone was being widely used off-label at 3 months in an attempt to increase milk supply– sometimes unsuccessfully, since many NBFW at the 3-month time point reported using domperidone to combat lactation disorders prior to stopping breastfeeding.

The pattern of medication use based on therapeutic category differed between BFW and NBFW in the CHILD Cohort study (Fig. 1). This information is important to signal potential therapeutic areas where patients may be less likely to initiate or sustain breastfeeding as an example non-breastfeeding women used significantly more psychotropic medications than non-breastfeeding medications.

Our results were comparable to other studies that found that vitamins were more frequently used by BFW as was recommended [17, 21]. Most healthcare providers recommend continuation of prenatal vitamins during lactation which may also explain these findings. Differences were identified in rates of multivitamins across the study sites with highest use in Vancouver at 3 and 6 months postpartum. This could be explained by previous coverage available for some vulnerable populations through programs such as BC Employment and Assistance in Vancouver until seven months postpartum while no coverage is found that specifically tailors to lactating women in Manitoba, Edmonton or Toronto [22–24]. These results might reflect the effectiveness of a coverage program for prenatal findings in the postnatal period. Differences in medication were also observed between sites, for example domperidone was used the most in Vancouver at 3 months and norethisterone was used the most in Edmonton at 12 months. It was unclear why this pattern existed.

Our analysis showed that BFW used psychotropic medications at a rate lower (4.3% at 3 months postpartum) than the general population while NBFW used these agents at a rate closer to the general population (11.2% at 3 months postpartum). According to the 2002 Canadian census, the rate of psychotropic medication use in Canadian women age 15 years and older was 9.5% [25]. This pattern is a complex issue which could be explained by recent literature showing woman taking psychotropic agents are less likely to initiate breastfeeding or have higher rate of breastfeeding cessation [26]. Other studies also found that psychotropic medications were more commonly used by NBFW [17]. A previous study reported that about 16.9% women who received psychotropic medication discontinued breastfeeding although in most cases it was not due to psychiatrists recommendation or adverse events due to medications [27].

In addition to this, at 3 months specifically, significantly more NBFW used opioid analgesics than BFW. Opioid analgesics transfer into maternal milk; medications such as fentanyl and propoxyphene are considered safe during lactation while codeine, tramadol, morphine, hydromorphone, methadone and oxycodone are considered moderately safe during breastfeeding [28]. The most commonly used oral-contraceptives for all women postpartum was norethisterone (a progestin-only contraceptive) which is predictable since it has been shown that the estrogen component of combined oral contraceptives may cause a decline in breast milk volume [29]. A previous study showed women taking progestogen-only contraception were less likely to stop breastfeeding before 6 months postpartum compared to combined contraception with estrogen [29]. This could explain the higher use of norethisterone in BFW than NBFW in our analysis. In this cohort, hormonal contraceptives were reported more commonly in women who were not breastfeeding which has been reported in other studies [17]. Levothyroxine was among the most common prescription medications used by both BFW and NBFW. The indication for levothyroxine is thyroid dysfunction such as postpartum thyroiditis which has a prevalence of approximately 8.1% according to studies done in the US [30]. Mothers in this cohort appear to be using the medication in accordance with this indication as the majority of mothers self-reported using levothyroxine to combat hypothyroidism.

The most commonly used anti-infective at 3 months postpartum was cephalexin, with no significant difference in cephalexin use between BFW and NBFW in the first 3 months postpartum which could be indicated for treating mastitis or breast abscess associated with breastfeeding. Mothers self-reported using antibiotics for mastitis, unspecific infections, and other breast abnormalities related to lactation which likely contributed to the increased number of BFW taking antibiotics. Approximately 75–95% of cases of mastitis occur before the infant is 3 months of age and cephalexin is a common treatment option [31]. On the other hand, the overall similar rates of cephalexin use between breastfeeding and non-breastfeeding women at 3 months could also indicate that non-breastfeeding women taking antibiotics could have stopped breastfeeding due to mastitis. There is evidence revealing that women with mastitis have poorer breastfeeding outcomes and higher rates of abrupt breastfeeding cessation although whether this relates to mastitis itself of use of antibiotics is unclear [32].At 6 and 12 months postpartum, vitamin/minerals and non-opioid analgesics were the most commonly used categories by BFW. This is similar to findings in a Brazilian study which evaluated medication use during the first 12 months and found that the most commonly used class of medications were analgesics/antipyretics, iron preparations and NSAIDs [4, 17]. Our study supports this as the most common non-prescription medications were a variety of vitamins/minerals, analgesics such as acetaminophen or ibuprofen at 3, 6 and 12 months postpartum. A study in Jordan done in 2015, also similarly found that the most commonly used medications were analgesics followed by antibiotics [33]. Another study by Hale et al., reported that common medications used in the postpartum period include analgesics, antihypertensives, sedatives, antidepressants, antipsychotics, antiepileptics, antibiotics, and steroids [5]. Our analysis partially aligns with anti-infectives being the most common therapeutic category used at 3 months (after supplements/herbal products and vitamins/minerals) and analgesics being the most common at 6 and 12 months (after vitamins/minerals) among BFW.

### Strengths and limitations

The main strength of our study is the inclusion of pattern of use of NPM which included over-the-counter medications and other medications allowing us to analyze the use of products that are not available as part of provincial administrative health data (e.g. DPIN prescribing records). One of the main limitations is the accuracy of self-reported medication use and the timing of medication use and breastfeeding reporting. As we were not able to compare the exact breastfeeding stop date and medication exposure date, misclassification of non-breastfeeding women is possible. For example, it is uncertain whether some women might have stopped breastfeeding to take certain medications or were taking certain medications that resulted in breastfeeding difficulties and earlier breastfeeding discontinuation. Further studies are required to explore potential sources of confounding. Another limitation is that there was overlap of prescription medications and non-prescription medications under most therapeutic categories. As an example, under the category ‘anti-allergy’ medications, there might be prescription and non-prescription medications. Under-reporting of medication use by mothers may occur for various reasons, including recall bias. A study examined validity of questionnaires about medication use during pregnancy by comparing with pharmacy records, and found the questionnaire had 76% sensitivity when a question was asked in an indication-oriented fashion, however some medications were mentioned that were missed by pharmacy records [34]. In the CHILD questionnaire, women were given examples of common indications for medications in order to aid in the recall of the mothers about their medication use. Another limitation is the presence of selection bias and response bias where the mother-infant dyads might not be representative of the whole Canadian population. Women who had a higher education level who had at least some college or university experience (93.6%) were over-represented in this analysis compared to the general population, as were women who initiated breastfeeding in hospital (96.5%) which was higher than the Canadian average (89%), and therefore our results may not be generalizable at the population level [35]. Another limitation is that indications for medication use questionnaires utilized free text and therefore many mothers did not respond which does not provide an accurate representation of all the indications for medication use.

Although, there have been more recent studies on postpartum medication use which were mentioned above and aligned with our results, it is important to continue to evaluate medication use in women postpartum to assess continued relevancy of our analysis in the Canadian population. To our knowledge this is the largest, curated and ontologized dataset of self-reported “reasons for medication use” for BFW. While limited, since response was optional, this does provide some insight into the perceptions and rationale behind medication use, which would not be available otherwise. In particular, it provided important confirmation of off-label domperidone use in the breastfeeding and non-breastfeeding population. This study supports further research into the prevalence and effectiveness of domperidone prescribed for insufficient lactation.

Epidemiological data on medication use patterns and reasons for use during lactation is key to informing public health policies and clinical care guidelines. This analysis collectively provides an important description of medications and supplements being used during breastfeeding, along with ontologized reasons for use, which lays the groundwork for future studies to enable healthcare providers and breastfeeding mothers to make evidence-informed decisions regarding the risk of discontinuation of a medication, safety of alternative medications as well as risk of exposures to medications/supplements during lactation on maternal-child health.

**Table 1:** Sociodemographic characteristics of breastfeeding and non-breastfeeding mothers in the CHILD cohort study at 3, 6 and 12 months postpartum

	3 months			6 months			12 months		
	Breastfeeding women (*n* = 2540)	Non-breastfeeding women (*n* = 366)		Breastfeeding women (*n* = 1984)	Non-breastfeeding women (*n* = 639)		Breastfeeding women (*n* = 1180)	Non-breastfeeding women (*n* = 1413)	
	n (%)	n (%)	p	n (%)	n (%)	p	n (%)	n (%)	p
**Country of birth** **Canadian-born**	1888 (74%)	274 (75%)	NS	1475 (76%)	509 (80%)	0.02	878 (74%)	1104 (78%)	0.02
**Foreign-born**	641 (25%)	86 (23%)		465 (24%)	122 (19%)		296 (25%)	300 (21%)	
**No answer**	11 (0.4%)	6 (2%)		8 (0.4%)	8 (1%)		6 (1%)	9 (1%)	
**Age at delivery (yr)** **<30**	665 (26%)	163 (45%)	< 0.001	504 (26%)	250 (39%)	< 0.001	261 (22%)	501 (35%)	< 0.001
**30–35**	1126 (44%)	111 (30%)		861 (44%)	252 (39%)		530 (45%)	577 (41%)	
**>35**	749 (29%)	83 (23%)		583 (30%)	137 (21%)		389 (33%)	335 (24%)	
**Education** **Not completed HS**	665 (26%)	163 (45%)	< 0.001	504 (26%)	250 (39%)	< 0.001	261 (22%)	501 (35%)	< 0.001
**Completed HS**	1126 (44%)	111 (30%)		861 (44%)	252 (39%)		530 (45%)	577 (41%)	
**Some college/university**	749 (29%)	83 (23%)		583 (30%)	137 (21%)		389 (33%)	335 (24%)	
**Completed college/university**	665 (26%)	163 (45%)		504 (26%)	250 (39%)		261 (22%)	501 (35%)	
**Masters/PhD**	1126 (44%)	111 (30%)		861 (44%)	252 (39%)		530 (45%)	577 (41%)	
**No answer**	749 (29%)	83 (23%)		583 (30%)	137 (21%)		389 (33%)	335 (24%)	
**Annual household income** **<50 K**	266 (10%)	71 (19%)	< 0.001	193 (10%)	106 (17%)	< 0.001	110 (9%)	187 (13%)	0.003
**50-100 K**	751 (30%)	104 (28%)		596 (31%)	187 (29%)		390 (33%)	401 (28%)	
**100-150 K**	661 (26%)	83 (23%)		507 (26%)	151 (24%)		308 (26%)	354 (25%)	
**>150 K**	581 (23%)	58 (16%)		438 (22%)	113 (18%)		238 (20%)	308 (22%)	
**No answer**	277 (11%)	50 (14%)		210 (11%)	82 (13%)		133 (11%)	160 (11%)	
**Study Centre** **Edmonton**	521 (21%)	109 (30%)	< 0.001	401 (21%)	166 (26%)	< 0.001	211 (18%)	386 (27%)	< 0.001
**Toronto**	613 (24%)	89 (24%)		436 (22%)	135 (21%)		209 (18%)	300 (21%)	
**Vancouver**	661 (26%)	43 (12%)		503 (26%)	82 (13%)		400 (34%)	233 (16%)	
**Manitoba**	745 (29%)	125 (34%)		608 (31%)	256 (40%)		360 (31%)	494 (35%)	

**Table 2:** 10 most common prescription medications used by mothers while breastfeeding during the first 3 months postpartum (*n* = 2540) compared to non-breastfeeding mothers (*n* = 366) with most common reasons for medication use in the CHILD cohort study

	Breastfeeding mothers			Non-breastfeeding mothers		
Prescription medication	Reported reason for use*	Mothers who used medication for specified reason.		Reported reason for use*	Mothers who used medication for specified reason.	
		%	n		%	n
domperidone	**Lactation disorder**	97.25	212	**Lactation disorder**	100	44
	Gastroesophageal reflux	1.38	3			
norethisterone	Contraception	99.22	127	Contraception	100	4
levothyroxine	Hypothyroidism	97.22	105	Hypothyroidism	88.89	16
cephalexin	Mastitis	41.57	37	Mastitis	18.8	2
	Infection	16.85	13			
	Breast infection	8.99	8			
amoxicillin	Infection	16.92	11	No data		
	Urinary tract infection	10.77	7			
	Bladder Infection	10.77	7			
	Mastitis	9.23	6			
salbutamol	Asthma	79.25	42	Asthma	70	7
	Chest infection	3.77	2	Bronchitis	20	2
betamethasone;clotrimazole;miconazole;mupirocin	Reason not given	95.92	47	Reason not given	100	
antibacterial drug name unknown	Reason not given	100	41			
cloxacillin	Mastitis	52.94	16	No data		
	Infection	23.53	8			
	Infection at incision site	5.88	2			
nystatin	Candidiasis prevention	42.42	14	No data		
	Candidiasis infection	18.18	6			
	Fungal infection	15.15	5			
	Nipple abnormality	9.09	3			

* Reason for medication use was ontologized (assigned an ontology/controlled vocabulary with hierarchy)

**The combination of medications used and indication that was only used once was removed from the table

***Indications related to lactation were bolded

**Table 3:** 10 most common prescription medications used by mothers while breastfeeding at 6 months (*n* = 1948) compared to non-breastfeeding mothers (*n* = 639) with most common reasons for medication use in the CHILD cohort study

	Breastfeeding mothers			Non-breastfeeding mothers		
**Prescription medication**	Reported reason for use*	**Mothers who used medication for specified reason**		Reported reason for use*	**Mothers who used medication for specified reason**	
		**%**	**n**		**%**	**n**
domperidone	Lactation disorder	98.15	106	Lactation disorder	88.89	8
norethisterone	Contraception	100	84	Contraception	100	9
levothyroxine	Hypothyroidism	97.44	76	Hypothyroidism	88	22
amoxicillin	Sinusitis	21.43	6	No Data		
	Urinary tract infection	10.71	3			
	Pharyngitis	10.71	3			
	Respiratory tract infection	7.14	2			
	Ear infection	7.14	2			
citalopram	Depression	77.78	21	Depression	81.82	9
	Anxiety	37.04	10	Anxiety	36.36	4
salbutamol	Asthma	80.77	21	Asthma	88.89	8
cephalexin	Mastitis	44.44	8	No data		
	Breast infection	11.11	2			
	Infection	11.11	2			
antibacterial drug name unknown	Reason not given	100	17	Reason not given	100	3
sertraline	Depression	58.82	10	Depression	88.89	8
	Anxiety	41.18	7	Anxiety	33.33	3
	Postpartum depression	11.76	2			
hydrocortisone	Eczema	38.46	5	No data		

* Reason for medication use was ontologized (assigned an ontology/controlled vocabulary with hierarchy)

**The combination of medications used and indication that was only used once was removed from the table

***Indications related to lactation were bolded

**Table 4:** 10 most common prescription medications used by mothers while breastfeeding at 12 months (*n* = 1180) compared to non-breastfeeding mothers (*n* = 1413) with most common reasons for medication use in the CHILD cohort study

	Breastfeeding mothers			Non-breastfeeding mothers		
**Prescription medication**	Reported reason for use*	**Mothers who used medication for specified reason**		Reported Reason for Use*	**Mothers who used medication for specified reason**	
		**%**	**n**		**%**	**n**
norethisterone	Contraception	100	48	Contraception	100	28
levothyroxine	Hypothyroidism	97.37	37	Hypothyroidism	94.29	66
domperidone	Lactation Disorder	86.84	33	Lactation disorder	83.33	15
	Nausea	5.26	2			
amoxicillin	Infection	18.92	7	Infection	18.92	7
	Ear Infection	16.22	6	Sinusitis	16.22	6
	Sinusitis	10.81	4	Pharyngitis	13.51	5
	Respiratory tract infection	5.41	2	Ear infection	8.11	3
salbutamol	Asthma	89.29	25	Asthma	90.63	29
	Common cold	10.71	3			
citalopram	Depression	69.57	16	Depression	90	27
	Anxiety	43.48	10	Anxiety	26.67	8
sertraline	Depression	58.82	10	Depression	76.92	10
	Anxiety	23.53	4	Anxiety	15.38	2
betamethasone	Eczema	71.43	10	Skin rash	28.57	2
	Atopic dermatitis	14.29	2	Eczema	28.57	2
antibacterial drug	Pharyngitis	15.38	2	Urinary tract infection	30.77	4
cephalexin	Mastitis	66.67	8	Infection	25	2

* Reason for medication use was ontologized (assigned an ontology/controlled vocabulary with hierarchy)

**The combination of medications used and indication that was only used once was removed from the table

***Indications related to lactation were bolded

### Electronic supplementary material

Below is the link to the electronic supplementary material.

Supplementary Material 1

## Data Availability

Data available within the article or its additional files.
