# Supplementary material for: The use of prescription medications and non-prescription medications during lactation in a prospective Canadian cohort study
Source: Int Breastfeed J. 2024 Apr 8;19:23. doi: 10.1186/s13006-024-00628-x (PMC11000278; doi:10.1186/s13006-024-00628-x)
Supplement: Supplementary file 1 — Supplementary Material 1 [file 13006_2024_628_MOESM1_ESM.docx]

**Additional file 1. Most common prescription medications and non-prescription medications use by breastfeeding women at 3 (n=2540), 6**  **(n=1948)**  **and 12 months (n=1180)**  **postpartum by site**

**
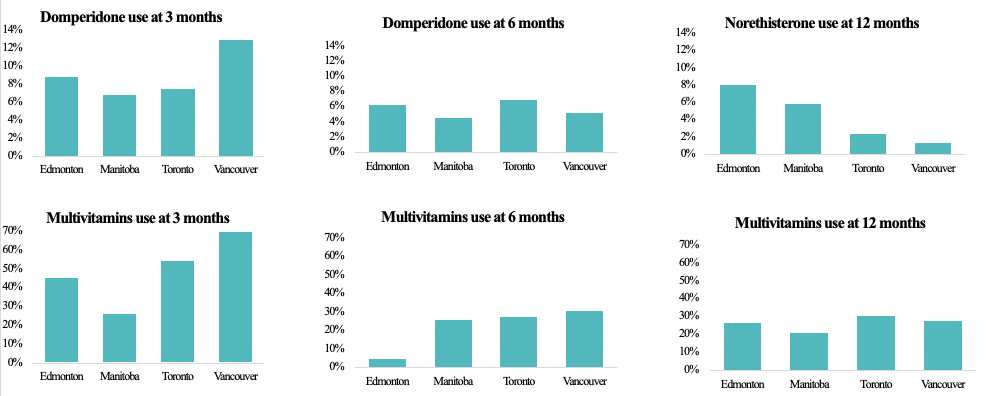
**

*

*

*

*

* denotes statistical significance

**Additional File 2. List of all prescription medications and usage by breastfeeding and non-breastfeeding women at least once at 3, 6 and 12 months postpartum**

| **All Prescription medications (curated generic names)** | **N Women Breastfeeding at 3mos (n=2540)** | **%** | **N Women Non-breastfeeding at 3mos (n=366)** | **%** | **N Women Breastfeeding at 6mos (n=1984)** | **%** | **N Women Non-breastfeeding at 6mos (n=639)** | **%** | **N Women Breastfeeding at 12mos (n=1180)** | **%** | **N Women Non-breastfeeding at 12mos (n=1413)** | **%** |
| --- | --- | --- | --- | --- | --- | --- | --- | --- | --- | --- | --- | --- |
| acetaminophen;caffeine;codeine | 31 | 1.2% | 6 | 1.6% | 10 | 0.5% | 6 | 0.9% | 1 | 0.1% | 0 | 0.0% |
| acetaminophen;codeine | 4 | 0.2% | 4 | 1.1% | 0 | 0.0% | 0 | 0.0% | 1 | 0.1% | 4 | 0.3% |
| acetaminophen;codeine;caffeine | 0 | 0.0% | 0 | 0.0% | 0 | 0.0% | 0 | 0.0% | 9 | 0.8% | 22 | 1.6% |
| acetaminophen;codeine;doxylamine | 0 | 0.0% | 0 | 0.0% | 0 | 0.0% | 1 | 0.2% | 0 | 0.0% | 0 | 0.0% |
| acetaminophen;doxylamine succinate;codeine | 0 | 0.0% | 0 | 0.0% | 0 | 0.0% | 1 | 0.2% | 0 | 0.0% | 1 | 0.1% |
| acetaminophen;hydrocodone | 0 | 0.0% | 0 | 0.0% | 0 | 0.0% | 0 | 0.0% | 0 | 0.0% | 1 | 0.1% |
| acetaminophen;oxycodone | 1 | 0.0% | 1 | 0.3% | 1 | 0.1% | 2 | 0.3% | 1 | 0.1% | 1 | 0.1% |
| acetaminophen;tramadol | 0 | 0.0% | 0 | 0.0% | 0 | 0.0% | 2 | 0.3% | 0 | 0.0% | 4 | 0.3% |
| acyclovir | 5 | 0.2% | 2 | 0.5% | 2 | 0.1% | 0 | 0.0% | 3 | 0.3% | 0 | 0.0% |
| adapalene | 0 | 0.0% | 0 | 0.0% | 0 | 0.0% | 0 | 0.0% | 1 | 0.1% | 0 | 0.0% |
| adapalene;benzoyl peroxide | 0 | 0.0% | 0 | 0.0% | 0 | 0.0% | 1 | 0.2% | 0 | 0.0% | 0 | 0.0% |
| albuterol | 0 | 0.0% | 0 | 0.0% | 0 | 0.0% | 0 | 0.0% | 1 | 0.1% | 0 | 0.0% |
| allopurinol | 0 | 0.0% | 0 | 0.0% | 0 | 0.0% | 0 | 0.0% | 0 | 0.0% | 1 | 0.1% |
| alprazolam | 0 | 0.0% | 0 | 0.0% | 0 | 0.0% | 1 | 0.2% | 1 | 0.1% | 1 | 0.1% |
| amcinonide | 0 | 0.0% | 0 | 0.0% | 0 | 0.0% | 0 | 0.0% | 0 | 0.0% | 1 | 0.1% |
| aminoglycoside antibiotic;betamethasone;gentamicin | 1 | 0.0% | 0 | 0.0% | 0 | 0.0% | 0 | 0.0% | 0 | 0.0% | 0 | 0.0% |
| aminosalicylic acid | 1 | 0.0% | 0 | 0.0% | 0 | 0.0% | 0 | 0.0% | 0 | 0.0% | 0 | 0.0% |
| amitriptyline | 0 | 0.0% | 0 | 0.0% | 0 | 0.0% | 0 | 0.0% | 0 | 0.0% | 5 | 0.4% |
| amlodipine | 1 | 0.0% | 0 | 0.0% | 0 | 0.0% | 0 | 0.0% | 0 | 0.0% | 0 | 0.0% |
| amoxicillin | 68 | 2.7% | 7 | 1.9% | 28 | 1.4% | 4 | 0.6% | 37 | 3.1% | 41 | 2.9% |
| amoxicillin;clarithromycin;lansoprazole | 0 | 0.0% | 0 | 0.0% | 0 | 0.0% | 0 | 0.0% | 0 | 0.0% | 1 | 0.1% |
| amoxicillin;clavulanate potassium | 1 | 0.0% | 0 | 0.0% | 0 | 0.0% | 0 | 0.0% | 2 | 0.2% | 0 | 0.0% |
| amoxicillin;clavulanic acid | 3 | 0.1% | 0 | 0.0% | 2 | 0.1% | 1 | 0.2% | 6 | 0.5% | 2 | 0.1% |
| anaesthetic | 1 | 0.0% | 0 | 0.0% | 0 | 0.0% | 0 | 0.0% | 3 | 0.3% | 3 | 0.2% |
| anti-asthmatic drug | 1 | 0.0% | 0 | 0.0% | 0 | 0.0% | 0 | 0.0% | 0 | 0.0% | 0 | 0.0% |
| antibacterial drug | 0 | 0.0% | 0 | 0.0% | 0 | 0.0% | 0 | 0.0% | 13 | 1.1% | 16 | 1.1% |
| antibacterial drug name unknown | 43 | 1.7% | 11 | 3.0% | 17 | 0.9% | 3 | 0.5% | 0 | 0.0% | 0 | 0.0% |
| anticoagulant | 2 | 0.1% | 0 | 0.0% | 0 | 0.0% | 0 | 0.0% | 0 | 0.0% | 0 | 0.0% |
| antiemetic | 1 | 0.0% | 0 | 0.0% | 0 | 0.0% | 0 | 0.0% | 0 | 0.0% | 0 | 0.0% |
| antipsoriatic | 0 | 0.0% | 0 | 0.0% | 0 | 0.0% | 0 | 0.0% | 1 | 0.1% | 0 | 0.0% |
| antiviral drug | 0 | 0.0% | 0 | 0.0% | 0 | 0.0% | 0 | 0.0% | 0 | 0.0% | 1 | 0.1% |
| aripiprazole | 0 | 0.0% | 0 | 0.0% | 0 | 0.0% | 0 | 0.0% | 1 | 0.1% | 1 | 0.1% |
| atenolol | 0 | 0.0% | 1 | 0.3% | 0 | 0.0% | 0 | 0.0% | 0 | 0.0% | 1 | 0.1% |
| atorvastatin | 0 | 0.0% | 1 | 0.3% | 0 | 0.0% | 1 | 0.2% | 0 | 0.0% | 1 | 0.1% |
| atropine | 1 | 0.0% | 0 | 0.0% | 0 | 0.0% | 0 | 0.0% | 0 | 0.0% | 0 | 0.0% |
| azathioprine | 2 | 0.1% | 0 | 0.0% | 0 | 0.0% | 0 | 0.0% | 0 | 0.0% | 3 | 0.2% |
| azelaic acid | 0 | 0.0% | 0 | 0.0% | 0 | 0.0% | 0 | 0.0% | 1 | 0.1% | 0 | 0.0% |
| azithromycin | 3 | 0.1% | 0 | 0.0% | 5 | 0.3% | 5 | 0.8% | 7 | 0.6% | 10 | 0.7% |
| beclomethasone | 2 | 0.1% | 0 | 0.0% | 1 | 0.1% | 0 | 0.0% | 1 | 0.1% | 2 | 0.1% |
| benzydamine hydrochloride | 0 | 0.0% | 0 | 0.0% | 0 | 0.0% | 0 | 0.0% | 1 | 0.1% | 0 | 0.0% |
| benzyl benzoate;bismuth oxide;bismuth subgallate;hydrocortisone;peru balsam;zinc oxide | 2 | 0.1% | 0 | 0.0% | 1 | 0.1% | 0 | 0.0% | 0 | 0.0% | 0 | 0.0% |
| betahistine | 1 | 0.0% | 0 | 0.0% | 0 | 0.0% | 0 | 0.0% | 0 | 0.0% | 1 | 0.1% |
| betamethasone | 18 | 0.7% | 3 | 0.8% | 11 | 0.6% | 2 | 0.3% | 15 | 1.3% | 7 | 0.5% |
| betamethasone valerate | 4 | 0.2% | 0 | 0.0% | 2 | 0.1% | 0 | 0.0% | 3 | 0.3% | 0 | 0.0% |
| betamethasone;clotrimazole | 1 | 0.0% | 0 | 0.0% | 0 | 0.0% | 1 | 0.2% | 1 | 0.1% | 1 | 0.1% |
| betamethasone;clotrimazole;miconazole;mupirocin | 49 | 1.9% | 0 | 0.0% | 8 | 0.4% | 0 | 0.0% | 0 | 0.0% | 0 | 0.0% |
| betamethasone;mupirocin | 0 | 0.0% | 0 | 0.0% | 1 | 0.1% | 0 | 0.0% | 0 | 0.0% | 0 | 0.0% |
| betamethasone;salicylic acid | 1 | 0.0% | 0 | 0.0% | 2 | 0.1% | 0 | 0.0% | 0 | 0.0% | 0 | 0.0% |
| betaxolol | 1 | 0.0% | 0 | 0.0% | 0 | 0.0% | 0 | 0.0% | 1 | 0.1% | 1 | 0.1% |
| bisoprolol | 0 | 0.0% | 0 | 0.0% | 1 | 0.1% | 0 | 0.0% | 1 | 0.1% | 1 | 0.1% |
| bleomycin | 0 | 0.0% | 0 | 0.0% | 0 | 0.0% | 0 | 0.0% | 0 | 0.0% | 1 | 0.1% |
| bromocriptine | 0 | 0.0% | 0 | 0.0% | 0 | 0.0% | 0 | 0.0% | 0 | 0.0% | 1 | 0.1% |
| budesonide | 15 | 0.6% | 0 | 0.0% | 7 | 0.4% | 0 | 0.0% | 7 | 0.6% | 3 | 0.2% |
| budesonide;formoterol | 19 | 0.7% | 2 | 0.5% | 10 | 0.5% | 4 | 0.6% | 8 | 0.7% | 15 | 1.1% |
| buprenorphine;naloxone | 1 | 0.0% | 0 | 0.0% | 1 | 0.1% | 0 | 0.0% | 1 | 0.1% | 0 | 0.0% |
| bupropion | 7 | 0.3% | 6 | 1.6% | 4 | 0.2% | 7 | 1.1% | 2 | 0.2% | 16 | 1.1% |
| caffeine;codeine;doxylamine succinate | 0 | 0.0% | 0 | 0.0% | 0 | 0.0% | 0 | 0.0% | 0 | 0.0% | 1 | 0.1% |
| calcipotriol;betamethasone | 0 | 0.0% | 1 | 0.3% | 0 | 0.0% | 1 | 0.2% | 0 | 0.0% | 0 | 0.0% |
| candesartan | 0 | 0.0% | 1 | 0.3% | 0 | 0.0% | 1 | 0.2% | 0 | 0.0% | 0 | 0.0% |
| cannabinoid | 0 | 0.0% | 0 | 0.0% | 0 | 0.0% | 0 | 0.0% | 1 | 0.1% | 0 | 0.0% |
| carbamazepine | 2 | 0.1% | 2 | 0.5% | 2 | 0.1% | 1 | 0.2% | 1 | 0.1% | 3 | 0.2% |
| carbamazepine;cephalexin | 0 | 0.0% | 0 | 0.0% | 0 | 0.0% | 0 | 0.0% | 0 | 0.0% | 1 | 0.1% |
| cardiovascular drug name unknown | 1 | 0.0% | 0 | 0.0% | 0 | 0.0% | 0 | 0.0% | 0 | 0.0% | 0 | 0.0% |
| cefazolin | 1 | 0.0% | 0 | 0.0% | 0 | 0.0% | 0 | 0.0% | 0 | 0.0% | 0 | 0.0% |
| cefixime | 4 | 0.2% | 1 | 0.3% | 0 | 0.0% | 0 | 0.0% | 0 | 0.0% | 1 | 0.1% |
| cefprozil | 1 | 0.0% | 0 | 0.0% | 0 | 0.0% | 0 | 0.0% | 1 | 0.1% | 0 | 0.0% |
| ceftriaxone | 1 | 0.0% | 0 | 0.0% | 0 | 0.0% | 0 | 0.0% | 0 | 0.0% | 1 | 0.1% |
| cefuroxime | 3 | 0.1% | 0 | 0.0% | 0 | 0.0% | 0 | 0.0% | 1 | 0.1% | 2 | 0.1% |
| celecoxib | 2 | 0.1% | 0 | 0.0% | 0 | 0.0% | 1 | 0.2% | 0 | 0.0% | 1 | 0.1% |
| cephalexin | 94 | 3.7% | 11 | 3.0% | 18 | 0.9% | 1 | 0.2% | 15 | 1.3% | 12 | 0.8% |
| chlorhexidine | 1 | 0.0% | 0 | 0.0% | 0 | 0.0% | 0 | 0.0% | 0 | 0.0% | 1 | 0.1% |
| chloroquine | 0 | 0.0% | 0 | 0.0% | 0 | 0.0% | 0 | 0.0% | 0 | 0.0% | 1 | 0.1% |
| chorionic gonadotropin | 0 | 0.0% | 0 | 0.0% | 0 | 0.0% | 0 | 0.0% | 0 | 0.0% | 1 | 0.1% |
| ciclesonide | 3 | 0.1% | 0 | 0.0% | 4 | 0.2% | 0 | 0.0% | 5 | 0.4% | 1 | 0.1% |
| ciclopirox | 3 | 0.1% | 0 | 0.0% | 2 | 0.1% | 2 | 0.3% | 2 | 0.2% | 1 | 0.1% |
| ciprofloxacin | 12 | 0.5% | 1 | 0.3% | 0 | 0.0% | 0 | 0.0% | 2 | 0.2% | 10 | 0.7% |
| ciprofloxacin;dexamethasone | 2 | 0.1% | 2 | 0.5% | 2 | 0.1% | 1 | 0.2% | 0 | 0.0% | 1 | 0.1% |
| citalopram | 30 | 1.2% | 8 | 2.2% | 27 | 1.4% | 11 | 1.7% | 25 | 2.1% | 31 | 2.2% |
| clarithromycin | 9 | 0.4% | 4 | 1.1% | 7 | 0.4% | 3 | 0.5% | 8 | 0.7% | 6 | 0.4% |
| clavulanate | 2 | 0.1% | 0 | 0.0% | 0 | 0.0% | 0 | 0.0% | 0 | 0.0% | 0 | 0.0% |
| clavulanic acid | 0 | 0.0% | 0 | 0.0% | 0 | 0.0% | 0 | 0.0% | 1 | 0.1% | 1 | 0.1% |
| clindamycin | 27 | 1.1% | 3 | 0.8% | 6 | 0.3% | 0 | 0.0% | 1 | 0.1% | 7 | 0.5% |
| clindamycin;benzoyl peroxide | 0 | 0.0% | 0 | 0.0% | 0 | 0.0% | 0 | 0.0% | 2 | 0.2% | 0 | 0.0% |
| clioquinol;flumethasone | 0 | 0.0% | 0 | 0.0% | 1 | 0.1% | 0 | 0.0% | 0 | 0.0% | 0 | 0.0% |
| clobazam | 1 | 0.0% | 0 | 0.0% | 1 | 0.1% | 0 | 0.0% | 0 | 0.0% | 1 | 0.1% |
| clobetasol | 4 | 0.2% | 0 | 0.0% | 1 | 0.1% | 0 | 0.0% | 4 | 0.3% | 2 | 0.1% |
| clobetasol propionate | 0 | 0.0% | 0 | 0.0% | 0 | 0.0% | 0 | 0.0% | 1 | 0.1% | 0 | 0.0% |
| clobetasol;salicylic acid | 0 | 0.0% | 0 | 0.0% | 0 | 0.0% | 0 | 0.0% | 0 | 0.0% | 1 | 0.1% |
| clomiphene | 0 | 0.0% | 0 | 0.0% | 0 | 0.0% | 0 | 0.0% | 0 | 0.0% | 3 | 0.2% |
| clomipramine | 1 | 0.0% | 0 | 0.0% | 1 | 0.1% | 0 | 0.0% | 1 | 0.1% | 0 | 0.0% |
| clonazepam | 0 | 0.0% | 1 | 0.3% | 1 | 0.1% | 3 | 0.5% | 0 | 0.0% | 3 | 0.2% |
| clopidogrel | 0 | 0.0% | 0 | 0.0% | 0 | 0.0% | 0 | 0.0% | 0 | 0.0% | 1 | 0.1% |
| clotrimazole;betamethasone;miconazole;mupirocin | 0 | 0.0% | 0 | 0.0% | 0 | 0.0% | 0 | 0.0% | 1 | 0.1% | 0 | 0.0% |
| clotrimazole;hydrocortisone | 1 | 0.0% | 0 | 0.0% | 1 | 0.1% | 0 | 0.0% | 0 | 0.0% | 0 | 0.0% |
| cloxacillin | 35 | 1.4% | 2 | 0.5% | 9 | 0.5% | 0 | 0.0% | 9 | 0.8% | 2 | 0.1% |
| codeine | 1 | 0.0% | 0 | 0.0% | 0 | 0.0% | 0 | 0.0% | 0 | 0.0% | 5 | 0.4% |
| codeine; guaifenesin;pseudoephedrine;triprolidine | 0 | 0.0% | 0 | 0.0% | 1 | 0.1% | 1 | 0.2% | 0 | 0.0% | 0 | 0.0% |
| codeine;guaifenesin;pheniramine | 1 | 0.0% | 0 | 0.0% | 0 | 0.0% | 0 | 0.0% | 1 | 0.1% | 0 | 0.0% |
| codeine;guaifenesin;pseudoephedrine;triprolidine | 0 | 0.0% | 0 | 0.0% | 0 | 0.0% | 0 | 0.0% | 0 | 0.0% | 1 | 0.1% |
| codeine;pseudoephedrine;triprolidine | 0 | 0.0% | 0 | 0.0% | 0 | 0.0% | 0 | 0.0% | 0 | 0.0% | 1 | 0.1% |
| contraceptive drug | 0 | 0.0% | 0 | 0.0% | 0 | 0.0% | 0 | 0.0% | 3 | 0.3% | 1 | 0.1% |
| contraceptive drug name unknown | 6 | 0.2% | 0 | 0.0% | 0 | 0.0% | 5 | 0.8% | 0 | 0.0% | 0 | 0.0% |
| corticosteroid | 1 | 0.0% | 0 | 0.0% | 0 | 0.0% | 0 | 0.0% | 0 | 0.0% | 0 | 0.0% |
| cyclobenzaprine | 0 | 0.0% | 4 | 1.1% | 0 | 0.0% | 3 | 0.5% | 0 | 0.0% | 2 | 0.1% |
| cyclosporine | 2 | 0.1% | 0 | 0.0% | 0 | 0.0% | 0 | 0.0% | 1 | 0.1% | 0 | 0.0% |
| cyproterone;ethinyl estradiol | 0 | 0.0% | 0 | 0.0% | 0 | 0.0% | 0 | 0.0% | 1 | 0.1% | 3 | 0.2% |
| dalteparin | 4 | 0.2% | 0 | 0.0% | 0 | 0.0% | 0 | 0.0% | 0 | 0.0% | 0 | 0.0% |
| desloratadine;pseudoephedrine | 0 | 0.0% | 0 | 0.0% | 0 | 0.0% | 0 | 0.0% | 0 | 0.0% | 1 | 0.1% |
| desogestrel;ethinyl estradiol | 0 | 0.0% | 5 | 1.4% | 3 | 0.2% | 11 | 1.7% | 3 | 0.3% | 19 | 1.3% |
| desoximetasone | 0 | 0.0% | 0 | 0.0% | 0 | 0.0% | 0 | 0.0% | 1 | 0.1% | 1 | 0.1% |
| desvenlafaxine | 0 | 0.0% | 1 | 0.3% | 1 | 0.1% | 0 | 0.0% | 1 | 0.1% | 2 | 0.1% |
| dexamethasone | 1 | 0.0% | 0 | 0.0% | 1 | 0.1% | 0 | 0.0% | 1 | 0.1% | 4 | 0.3% |
| dexamethasone sodium phosphate | 0 | 0.0% | 0 | 0.0% | 1 | 0.1% | 0 | 0.0% | 0 | 0.0% | 0 | 0.0% |
| dextroamphetamine | 0 | 0.0% | 0 | 0.0% | 0 | 0.0% | 2 | 0.3% | 0 | 0.0% | 3 | 0.2% |
| dextromethorphan;guaifenesin;pseudoephedrine | 0 | 0.0% | 0 | 0.0% | 0 | 0.0% | 0 | 0.0% | 0 | 0.0% | 2 | 0.1% |
| diacerein;meloxicam | 0 | 0.0% | 0 | 0.0% | 0 | 0.0% | 0 | 0.0% | 0 | 0.0% | 1 | 0.1% |
| diazepam | 0 | 0.0% | 0 | 0.0% | 0 | 0.0% | 0 | 0.0% | 0 | 0.0% | 1 | 0.1% |
| dibucaine;esculin;framycetin;hydrocortisone | 4 | 0.2% | 0 | 0.0% | 5 | 0.3% | 0 | 0.0% | 1 | 0.1% | 2 | 0.1% |
| diclofenac | 11 | 0.4% | 2 | 0.5% | 2 | 0.1% | 2 | 0.3% | 1 | 0.1% | 8 | 0.6% |
| diclofenac;misoprostol | 0 | 0.0% | 0 | 0.0% | 1 | 0.1% | 0 | 0.0% | 0 | 0.0% | 1 | 0.1% |
| dicloxacillin | 1 | 0.0% | 0 | 0.0% | 0 | 0.0% | 0 | 0.0% | 1 | 0.1% | 0 | 0.0% |
| dicyclomine;mefenamic acid | 0 | 0.0% | 0 | 0.0% | 1 | 0.1% | 0 | 0.0% | 0 | 0.0% | 0 | 0.0% |
| diflucortolone valerate | 1 | 0.0% | 0 | 0.0% | 0 | 0.0% | 0 | 0.0% | 1 | 0.1% | 0 | 0.0% |
| diltiazem | 2 | 0.1% | 0 | 0.0% | 1 | 0.1% | 0 | 0.0% | 2 | 0.2% | 1 | 0.1% |
| dimethyl ether;propane | 0 | 0.0% | 0 | 0.0% | 0 | 0.0% | 0 | 0.0% | 1 | 0.1% | 0 | 0.0% |
| domperidone | 229 | 9.0% | 47 | 12.8% | 109 | 5.5% | 9 | 1.4% | 38 | 3.2% | 18 | 1.3% |
| doxepin | 0 | 0.0% | 1 | 0.3% | 0 | 0.0% | 0 | 0.0% | 0 | 0.0% | 0 | 0.0% |
| doxorubicin | 0 | 0.0% | 0 | 0.0% | 0 | 0.0% | 0 | 0.0% | 0 | 0.0% | 1 | 0.1% |
| doxycycline | 1 | 0.0% | 1 | 0.3% | 0 | 0.0% | 1 | 0.2% | 0 | 0.0% | 1 | 0.1% |
| doxycycline hyclate | 0 | 0.0% | 0 | 0.0% | 0 | 0.0% | 0 | 0.0% | 0 | 0.0% | 1 | 0.1% |
| doxylamine;pyridoxine | 22 | 0.9% | 3 | 0.8% | 1 | 0.1% | 1 | 0.2% | 1 | 0.1% | 12 | 0.8% |
| drospirenone;ethinyl estradiol | 2 | 0.1% | 3 | 0.8% | 0 | 0.0% | 7 | 1.1% | 0 | 0.0% | 10 | 0.7% |
| drug cream | 0 | 0.0% | 0 | 0.0% | 2 | 0.1% | 0 | 0.0% | 2 | 0.2% | 1 | 0.1% |
| drug cream name unknown | 25 | 1.0% | 2 | 0.5% | 1 | 0.1% | 0 | 0.0% | 0 | 0.0% | 0 | 0.0% |
| duloxetine | 2 | 0.1% | 0 | 0.0% | 1 | 0.1% | 0 | 0.0% | 0 | 0.0% | 3 | 0.2% |
| enalapril | 2 | 0.1% | 0 | 0.0% | 1 | 0.1% | 0 | 0.0% | 0 | 0.0% | 0 | 0.0% |
| enoxaparin | 3 | 0.1% | 1 | 0.3% | 1 | 0.1% | 0 | 0.0% | 1 | 0.1% | 0 | 0.0% |
| epinephrine | 1 | 0.0% | 0 | 0.0% | 0 | 0.0% | 0 | 0.0% | 1 | 0.1% | 1 | 0.1% |
| erythromycin | 4 | 0.2% | 1 | 0.3% | 1 | 0.1% | 0 | 0.0% | 7 | 0.6% | 1 | 0.1% |
| escitalopram | 12 | 0.5% | 10 | 2.7% | 9 | 0.5% | 12 | 1.9% | 6 | 0.5% | 21 | 1.5% |
| esomeprazole | 3 | 0.1% | 1 | 0.3% | 1 | 0.1% | 3 | 0.5% | 1 | 0.1% | 4 | 0.3% |
| esomeprazole;naproxen | 0 | 0.0% | 0 | 0.0% | 0 | 0.0% | 0 | 0.0% | 0 | 0.0% | 1 | 0.1% |
| estradiol | 2 | 0.1% | 0 | 0.0% | 0 | 0.0% | 1 | 0.2% | 1 | 0.1% | 1 | 0.1% |
| estrogen | 1 | 0.0% | 1 | 0.3% | 0 | 0.0% | 0 | 0.0% | 0 | 0.0% | 0 | 0.0% |
| estrone sodium sulfate | 5 | 0.2% | 0 | 0.0% | 5 | 0.3% | 0 | 0.0% | 2 | 0.2% | 0 | 0.0% |
| ethinyl estradiol;ethynodiol diacetate | 0 | 0.0% | 1 | 0.3% | 0 | 0.0% | 1 | 0.2% | 0 | 0.0% | 3 | 0.2% |
| ethinyl estradiol;etonogestrel | 1 | 0.0% | 1 | 0.3% | 2 | 0.1% | 2 | 0.3% | 1 | 0.1% | 7 | 0.5% |
| ethinyl estradiol;ferrous fumarate;norethindrone | 0 | 0.0% | 0 | 0.0% | 0 | 0.0% | 0 | 0.0% | 0 | 0.0% | 3 | 0.2% |
| ethinyl estradiol;levonorgestrel | 8 | 0.3% | 12 | 3.3% | 4 | 0.2% | 17 | 2.7% | 5 | 0.4% | 34 | 2.4% |
| ethinyl estradiol;norelgestromin | 0 | 0.0% | 0 | 0.0% | 0 | 0.0% | 1 | 0.2% | 1 | 0.1% | 5 | 0.4% |
| ethinyl estradiol;norethindrone | 0 | 0.0% | 1 | 0.3% | 0 | 0.0% | 3 | 0.5% | 0 | 0.0% | 6 | 0.4% |
| ethinyl estradiol;norgestimate | 4 | 0.2% | 7 | 1.9% | 4 | 0.2% | 12 | 1.9% | 4 | 0.3% | 32 | 2.3% |
| famciclovir | 0 | 0.0% | 0 | 0.0% | 0 | 0.0% | 1 | 0.2% | 0 | 0.0% | 0 | 0.0% |
| fentanyl | 1 | 0.0% | 0 | 0.0% | 0 | 0.0% | 0 | 0.0% | 0 | 0.0% | 0 | 0.0% |
| fluconazole | 8 | 0.3% | 0 | 0.0% | 1 | 0.1% | 0 | 0.0% | 3 | 0.3% | 0 | 0.0% |
| fludrocortisone | 1 | 0.0% | 0 | 0.0% | 1 | 0.1% | 0 | 0.0% | 1 | 0.1% | 0 | 0.0% |
| fluocinolone | 1 | 0.0% | 0 | 0.0% | 0 | 0.0% | 0 | 0.0% | 0 | 0.0% | 0 | 0.0% |
| fluocinonide | 1 | 0.0% | 0 | 0.0% | 1 | 0.1% | 0 | 0.0% | 0 | 0.0% | 1 | 0.1% |
| fluorometholone | 0 | 0.0% | 0 | 0.0% | 0 | 0.0% | 0 | 0.0% | 1 | 0.1% | 1 | 0.1% |
| fluoxetine | 8 | 0.3% | 4 | 1.1% | 7 | 0.4% | 2 | 0.3% | 3 | 0.3% | 12 | 0.8% |
| fluticasone | 18 | 0.7% | 4 | 1.1% | 11 | 0.6% | 1 | 0.2% | 13 | 1.1% | 15 | 1.1% |
| fluticasone furoate | 5 | 0.2% | 0 | 0.0% | 3 | 0.2% | 1 | 0.2% | 4 | 0.3% | 3 | 0.2% |
| fluticasone;salmeterol | 6 | 0.2% | 2 | 0.5% | 6 | 0.3% | 1 | 0.2% | 6 | 0.5% | 5 | 0.4% |
| formoterol | 1 | 0.0% | 0 | 0.0% | 1 | 0.1% | 0 | 0.0% | 0 | 0.0% | 1 | 0.1% |
| formoterol;mometasone | 0 | 0.0% | 0 | 0.0% | 1 | 0.1% | 0 | 0.0% | 1 | 0.1% | 0 | 0.0% |
| frovatriptan | 0 | 0.0% | 0 | 0.0% | 0 | 0.0% | 0 | 0.0% | 1 | 0.1% | 0 | 0.0% |
| furosemide | 3 | 0.1% | 0 | 0.0% | 1 | 0.1% | 0 | 0.0% | 0 | 0.0% | 0 | 0.0% |
| fusidic acid | 7 | 0.3% | 0 | 0.0% | 2 | 0.1% | 0 | 0.0% | 3 | 0.3% | 2 | 0.1% |
| gabapentin | 2 | 0.1% | 0 | 0.0% | 0 | 0.0% | 1 | 0.2% | 0 | 0.0% | 0 | 0.0% |
| ganirelix | 0 | 0.0% | 0 | 0.0% | 0 | 0.0% | 0 | 0.0% | 0 | 0.0% | 1 | 0.1% |
| gatifloxacin | 0 | 0.0% | 0 | 0.0% | 0 | 0.0% | 0 | 0.0% | 0 | 0.0% | 1 | 0.1% |
| general anaesthetic | 0 | 0.0% | 1 | 0.3% | 1 | 0.1% | 0 | 0.0% | 0 | 0.0% | 1 | 0.1% |
| gentamycin | 2 | 0.1% | 0 | 0.0% | 0 | 0.0% | 0 | 0.0% | 0 | 0.0% | 0 | 0.0% |
| glatiramer | 0 | 0.0% | 0 | 0.0% | 0 | 0.0% | 0 | 0.0% | 0 | 0.0% | 1 | 0.1% |
| glucocorticoid | 0 | 0.0% | 0 | 0.0% | 0 | 0.0% | 0 | 0.0% | 0 | 0.0% | 1 | 0.1% |
| gramicidin;neomycin;nystatin;triamcinolone | 17 | 0.7% | 1 | 0.3% | 3 | 0.2% | 0 | 0.0% | 0 | 0.0% | 2 | 0.1% |
| halobetasol | 0 | 0.0% | 0 | 0.0% | 1 | 0.1% | 0 | 0.0% | 0 | 0.0% | 0 | 0.0% |
| heparin | 4 | 0.2% | 0 | 0.0% | 0 | 0.0% | 0 | 0.0% | 0 | 0.0% | 0 | 0.0% |
| histamine antagonist | 0 | 0.0% | 0 | 0.0% | 0 | 0.0% | 0 | 0.0% | 0 | 0.0% | 1 | 0.1% |
| hydrochlorothiazide | 0 | 0.0% | 0 | 0.0% | 0 | 0.0% | 1 | 0.2% | 1 | 0.1% | 1 | 0.1% |
| hydrocortisone | 23 | 0.9% | 3 | 0.8% | 13 | 0.7% | 3 | 0.5% | 8 | 0.7% | 6 | 0.4% |
| hydrocortisone;pramoxine | 3 | 0.1% | 0 | 0.0% | 0 | 0.0% | 0 | 0.0% | 0 | 0.0% | 0 | 0.0% |
| hydrocortisone;pramoxine;zinc sulfate | 1 | 0.0% | 0 | 0.0% | 0 | 0.0% | 0 | 0.0% | 0 | 0.0% | 0 | 0.0% |
| hydrocortisone;urea | 0 | 0.0% | 0 | 0.0% | 0 | 0.0% | 0 | 0.0% | 0 | 0.0% | 1 | 0.1% |
| hydrocortisone;zinc sulfate | 7 | 0.3% | 0 | 0.0% | 2 | 0.1% | 0 | 0.0% | 2 | 0.2% | 0 | 0.0% |
| hydromorphone | 4 | 0.2% | 0 | 0.0% | 0 | 0.0% | 0 | 0.0% | 1 | 0.1% | 4 | 0.3% |
| hydroxychloroquine | 5 | 0.2% | 0 | 0.0% | 1 | 0.1% | 3 | 0.5% | 1 | 0.1% | 6 | 0.4% |
| hydroxyzine | 2 | 0.1% | 0 | 0.0% | 0 | 0.0% | 0 | 0.0% | 0 | 0.0% | 1 | 0.1% |
| imipramine | 0 | 0.0% | 0 | 0.0% | 0 | 0.0% | 0 | 0.0% | 0 | 0.0% | 2 | 0.1% |
| imiquimod | 1 | 0.0% | 0 | 0.0% | 1 | 0.1% | 1 | 0.2% | 0 | 0.0% | 1 | 0.1% |
| immune globulin | 1 | 0.0% | 0 | 0.0% | 0 | 0.0% | 0 | 0.0% | 1 | 0.1% | 0 | 0.0% |
| indomethacin | 0 | 0.0% | 0 | 0.0% | 0 | 0.0% | 0 | 0.0% | 0 | 0.0% | 1 | 0.1% |
| infliximab | 0 | 0.0% | 1 | 0.3% | 0 | 0.0% | 1 | 0.2% | 0 | 0.0% | 1 | 0.1% |
| insulin | 2 | 0.1% | 0 | 0.0% | 1 | 0.1% | 1 | 0.2% | 0 | 0.0% | 1 | 0.1% |
| insulin (human) | 1 | 0.0% | 0 | 0.0% | 0 | 0.0% | 0 | 0.0% | 0 | 0.0% | 0 | 0.0% |
| insulin aspart | 3 | 0.1% | 0 | 0.0% | 1 | 0.1% | 0 | 0.0% | 1 | 0.1% | 0 | 0.0% |
| insulin glargine | 0 | 0.0% | 0 | 0.0% | 1 | 0.1% | 1 | 0.2% | 0 | 0.0% | 1 | 0.1% |
| insulin isophane | 2 | 0.1% | 0 | 0.0% | 1 | 0.1% | 0 | 0.0% | 0 | 0.0% | 1 | 0.1% |
| insulin isophane;insulin | 0 | 0.0% | 0 | 0.0% | 0 | 0.0% | 0 | 0.0% | 0 | 0.0% | 1 | 0.1% |
| insulin lispro | 3 | 0.1% | 1 | 0.3% | 1 | 0.1% | 0 | 0.0% | 0 | 0.0% | 1 | 0.1% |
| interferon beta-1a | 0 | 0.0% | 0 | 0.0% | 0 | 0.0% | 1 | 0.2% | 0 | 0.0% | 2 | 0.1% |
| ipratropium | 0 | 0.0% | 0 | 0.0% | 1 | 0.1% | 0 | 0.0% | 0 | 0.0% | 0 | 0.0% |
| ketoconazole | 2 | 0.1% | 0 | 0.0% | 1 | 0.1% | 0 | 0.0% | 2 | 0.2% | 1 | 0.1% |
| ketorolac | 4 | 0.2% | 1 | 0.3% | 1 | 0.1% | 0 | 0.0% | 1 | 0.1% | 3 | 0.2% |
| ketorolac tromethamine | 1 | 0.0% | 0 | 0.0% | 0 | 0.0% | 0 | 0.0% | 0 | 0.0% | 1 | 0.1% |
| L-tryptophan | 0 | 0.0% | 0 | 0.0% | 0 | 0.0% | 0 | 0.0% | 0 | 0.0% | 2 | 0.1% |
| labetalol | 32 | 1.3% | 7 | 1.9% | 6 | 0.3% | 3 | 0.5% | 1 | 0.1% | 8 | 0.6% |
| lamotrigine | 1 | 0.0% | 1 | 0.3% | 1 | 0.1% | 1 | 0.2% | 1 | 0.1% | 2 | 0.1% |
| lansoprazole | 3 | 0.1% | 0 | 0.0% | 4 | 0.2% | 0 | 0.0% | 2 | 0.2% | 0 | 0.0% |
| levocarnitine | 1 | 0.0% | 0 | 0.0% | 0 | 0.0% | 0 | 0.0% | 0 | 0.0% | 0 | 0.0% |
| levofloxacin | 0 | 0.0% | 0 | 0.0% | 1 | 0.1% | 0 | 0.0% | 0 | 0.0% | 1 | 0.1% |
| levomefolic acid | 0 | 0.0% | 0 | 0.0% | 1 | 0.1% | 0 | 0.0% | 0 | 0.0% | 0 | 0.0% |
| levonorgestrel | 5 | 0.2% | 0 | 0.0% | 8 | 0.4% | 1 | 0.2% | 4 | 0.3% | 4 | 0.3% |
| levothyroxine | 108 | 4.3% | 18 | 4.9% | 79 | 4.0% | 25 | 3.9% | 41 | 3.5% | 74 | 5.2% |
| levothyroxine;liothyronine | 0 | 0.0% | 0 | 0.0% | 0 | 0.0% | 0 | 0.0% | 1 | 0.1% | 0 | 0.0% |
| liothyronine | 1 | 0.0% | 0 | 0.0% | 0 | 0.0% | 0 | 0.0% | 1 | 0.1% | 0 | 0.0% |
| liraglutide | 0 | 0.0% | 0 | 0.0% | 0 | 0.0% | 0 | 0.0% | 0 | 0.0% | 1 | 0.1% |
| lisdexamfetamine | 0 | 0.0% | 0 | 0.0% | 0 | 0.0% | 0 | 0.0% | 0 | 0.0% | 1 | 0.1% |
| lisinopril | 0 | 0.0% | 0 | 0.0% | 0 | 0.0% | 1 | 0.2% | 0 | 0.0% | 1 | 0.1% |
| local anaesthetic | 0 | 0.0% | 0 | 0.0% | 0 | 0.0% | 0 | 0.0% | 1 | 0.1% | 0 | 0.0% |
| lorazepam | 5 | 0.2% | 5 | 1.4% | 6 | 0.3% | 6 | 0.9% | 6 | 0.5% | 12 | 0.8% |
| loteprednol etabonate | 1 | 0.0% | 0 | 0.0% | 0 | 0.0% | 0 | 0.0% | 0 | 0.0% | 0 | 0.0% |
| medroxyprogesterone | 5 | 0.2% | 1 | 0.3% | 3 | 0.2% | 2 | 0.3% | 1 | 0.1% | 3 | 0.2% |
| mefenamic acid | 0 | 0.0% | 0 | 0.0% | 0 | 0.0% | 1 | 0.2% | 0 | 0.0% | 1 | 0.1% |
| mefloquine | 0 | 0.0% | 0 | 0.0% | 0 | 0.0% | 0 | 0.0% | 1 | 0.1% | 0 | 0.0% |
| meloxicam | 0 | 0.0% | 0 | 0.0% | 0 | 0.0% | 1 | 0.2% | 0 | 0.0% | 0 | 0.0% |
| menotropins | 0 | 0.0% | 0 | 0.0% | 0 | 0.0% | 0 | 0.0% | 0 | 0.0% | 1 | 0.1% |
| mepivacaine hydrochloride | 0 | 0.0% | 0 | 0.0% | 0 | 0.0% | 0 | 0.0% | 0 | 0.0% | 1 | 0.1% |
| mesalamine | 5 | 0.2% | 1 | 0.3% | 4 | 0.2% | 0 | 0.0% | 6 | 0.5% | 2 | 0.1% |
| mesalazine | 0 | 0.0% | 1 | 0.3% | 0 | 0.0% | 1 | 0.2% | 0 | 0.0% | 1 | 0.1% |
| metformin | 2 | 0.1% | 1 | 0.3% | 0 | 0.0% | 3 | 0.5% | 1 | 0.1% | 4 | 0.3% |
| methadone | 0 | 0.0% | 1 | 0.3% | 0 | 0.0% | 0 | 0.0% | 0 | 0.0% | 1 | 0.1% |
| methimazole | 0 | 0.0% | 0 | 0.0% | 0 | 0.0% | 0 | 0.0% | 0 | 0.0% | 2 | 0.1% |
| methotrexate | 0 | 0.0% | 1 | 0.3% | 0 | 0.0% | 0 | 0.0% | 0 | 0.0% | 1 | 0.1% |
| methylphenidate | 1 | 0.0% | 0 | 0.0% | 0 | 0.0% | 0 | 0.0% | 1 | 0.1% | 1 | 0.1% |
| methylprednisolone | 0 | 0.0% | 0 | 0.0% | 0 | 0.0% | 0 | 0.0% | 1 | 0.1% | 0 | 0.0% |
| metoclopramide | 3 | 0.1% | 0 | 0.0% | 2 | 0.1% | 0 | 0.0% | 0 | 0.0% | 6 | 0.4% |
| metoprolol | 1 | 0.0% | 0 | 0.0% | 1 | 0.1% | 0 | 0.0% | 0 | 0.0% | 1 | 0.1% |
| metronidazole | 18 | 0.7% | 4 | 1.1% | 2 | 0.1% | 1 | 0.2% | 3 | 0.3% | 2 | 0.1% |
| metronidazole;nystatin | 1 | 0.0% | 0 | 0.0% | 0 | 0.0% | 1 | 0.2% | 1 | 0.1% | 0 | 0.0% |
| midodrine | 0 | 0.0% | 0 | 0.0% | 0 | 0.0% | 0 | 0.0% | 1 | 0.1% | 0 | 0.0% |
| minocycline | 0 | 0.0% | 0 | 0.0% | 0 | 0.0% | 0 | 0.0% | 1 | 0.1% | 2 | 0.1% |
| mirtazapine | 0 | 0.0% | 0 | 0.0% | 0 | 0.0% | 0 | 0.0% | 0 | 0.0% | 1 | 0.1% |
| misoprostol | 2 | 0.1% | 0 | 0.0% | 0 | 0.0% | 0 | 0.0% | 1 | 0.1% | 1 | 0.1% |
| mometasone | 6 | 0.2% | 1 | 0.3% | 1 | 0.1% | 1 | 0.2% | 3 | 0.3% | 5 | 0.4% |
| montelukast | 1 | 0.0% | 0 | 0.0% | 2 | 0.1% | 0 | 0.0% | 0 | 0.0% | 2 | 0.1% |
| morphine | 6 | 0.2% | 3 | 0.8% | 4 | 0.2% | 0 | 0.0% | 0 | 0.0% | 2 | 0.1% |
| moxifloxacin | 1 | 0.0% | 0 | 0.0% | 1 | 0.1% | 0 | 0.0% | 2 | 0.2% | 1 | 0.1% |
| nadolol | 0 | 0.0% | 0 | 0.0% | 0 | 0.0% | 0 | 0.0% | 0 | 0.0% | 1 | 0.1% |
| name unknown | 0 | 0.0% | 0 | 0.0% | 1 | 0.1% | 0 | 0.0% | 0 | 0.0% | 0 | 0.0% |
| naproxen | 20 | 0.8% | 5 | 1.4% | 6 | 0.3% | 4 | 0.6% | 4 | 0.3% | 10 | 0.7% |
| nifedipine | 16 | 0.6% | 3 | 0.8% | 4 | 0.2% | 2 | 0.3% | 0 | 0.0% | 3 | 0.2% |
| nitrofurantoin | 15 | 0.6% | 3 | 0.8% | 5 | 0.3% | 1 | 0.2% | 5 | 0.4% | 6 | 0.4% |
| norethindrone | 2 | 0.1% | 1 | 0.3% | 0 | 0.0% | 1 | 0.2% | 1 | 0.1% | 1 | 0.1% |
| norethindrone acetate;ethinyl estradiol | 0 | 0.0% | 0 | 0.0% | 0 | 0.0% | 0 | 0.0% | 0 | 0.0% | 1 | 0.1% |
| norethisterone | 128 | 5.0% | 4 | 1.1% | 84 | 4.2% | 9 | 1.4% | 48 | 4.1% | 28 | 2.0% |
| norfloxacin | 0 | 0.0% | 0 | 0.0% | 0 | 0.0% | 0 | 0.0% | 0 | 0.0% | 1 | 0.1% |
| nortriptyline | 0 | 0.0% | 0 | 0.0% | 0 | 0.0% | 0 | 0.0% | 1 | 0.1% | 0 | 0.0% |
| nystatin | 34 | 1.3% | 1 | 0.3% | 5 | 0.3% | 0 | 0.0% | 2 | 0.2% | 1 | 0.1% |
| ofloxacin | 0 | 0.0% | 0 | 0.0% | 0 | 0.0% | 0 | 0.0% | 1 | 0.1% | 0 | 0.0% |
| olanzapine | 0 | 0.0% | 2 | 0.5% | 1 | 0.1% | 1 | 0.2% | 0 | 0.0% | 6 | 0.4% |
| olopatadine | 0 | 0.0% | 0 | 0.0% | 1 | 0.1% | 0 | 0.0% | 1 | 0.1% | 3 | 0.2% |
| omeprazole | 4 | 0.2% | 0 | 0.0% | 2 | 0.1% | 1 | 0.2% | 4 | 0.3% | 6 | 0.4% |
| ondansetron | 1 | 0.0% | 0 | 0.0% | 0 | 0.0% | 0 | 0.0% | 0 | 0.0% | 4 | 0.3% |
| ophthalmology drug name unknown | 1 | 0.0% | 0 | 0.0% | 0 | 0.0% | 0 | 0.0% | 0 | 0.0% | 0 | 0.0% |
| oxcarbazepine | 1 | 0.0% | 0 | 0.0% | 1 | 0.1% | 0 | 0.0% | 0 | 0.0% | 1 | 0.1% |
| oxycodone | 9 | 0.4% | 6 | 1.6% | 1 | 0.1% | 1 | 0.2% | 2 | 0.2% | 4 | 0.3% |
| pantoprazole | 3 | 0.1% | 0 | 0.0% | 3 | 0.2% | 2 | 0.3% | 1 | 0.1% | 8 | 0.6% |
| paroxetine | 4 | 0.2% | 1 | 0.3% | 5 | 0.3% | 5 | 0.8% | 3 | 0.3% | 7 | 0.5% |
| pegfilgrastim | 0 | 0.0% | 0 | 0.0% | 0 | 0.0% | 0 | 0.0% | 0 | 0.0% | 1 | 0.1% |
| penbutolol | 1 | 0.0% | 0 | 0.0% | 0 | 0.0% | 0 | 0.0% | 0 | 0.0% | 0 | 0.0% |
| penicillin | 9 | 0.4% | 1 | 0.3% | 4 | 0.2% | 0 | 0.0% | 2 | 0.2% | 15 | 1.1% |
| phenazopyridine | 0 | 0.0% | 0 | 0.0% | 0 | 0.0% | 0 | 0.0% | 1 | 0.1% | 0 | 0.0% |
| phenoxymethylpenicillin | 3 | 0.1% | 0 | 0.0% | 2 | 0.1% | 0 | 0.0% | 0 | 0.0% | 3 | 0.2% |
| phenytoin | 1 | 0.0% | 0 | 0.0% | 1 | 0.1% | 0 | 0.0% | 1 | 0.1% | 0 | 0.0% |
| pimecrolimus | 0 | 0.0% | 0 | 0.0% | 0 | 0.0% | 0 | 0.0% | 1 | 0.1% | 0 | 0.0% |
| piperacillin | 1 | 0.0% | 0 | 0.0% | 0 | 0.0% | 0 | 0.0% | 0 | 0.0% | 0 | 0.0% |
| polyethylene glycol | 0 | 0.0% | 0 | 0.0% | 0 | 0.0% | 0 | 0.0% | 0 | 0.0% | 1 | 0.1% |
| polymyxin b sulfate;trimethoprim | 1 | 0.0% | 0 | 0.0% | 2 | 0.1% | 1 | 0.2% | 0 | 0.0% | 0 | 0.0% |
| pramocaine | 0 | 0.0% | 0 | 0.0% | 1 | 0.1% | 0 | 0.0% | 0 | 0.0% | 0 | 0.0% |
| pramoxine | 2 | 0.1% | 0 | 0.0% | 0 | 0.0% | 0 | 0.0% | 0 | 0.0% | 0 | 0.0% |
| prednisolone | 3 | 0.1% | 0 | 0.0% | 2 | 0.1% | 0 | 0.0% | 2 | 0.2% | 0 | 0.0% |
| prednisolone;sulfacetamide sodium | 1 | 0.0% | 0 | 0.0% | 0 | 0.0% | 0 | 0.0% | 0 | 0.0% | 0 | 0.0% |
| prednisone | 12 | 0.5% | 3 | 0.8% | 5 | 0.3% | 2 | 0.3% | 10 | 0.8% | 12 | 0.8% |
| pregabalin | 1 | 0.0% | 0 | 0.0% | 0 | 0.0% | 0 | 0.0% | 0 | 0.0% | 0 | 0.0% |
| prescription drug name unknown | 2 | 0.1% | 0 | 0.0% | 0 | 0.0% | 0 | 0.0% | 0 | 0.0% | 0 | 0.0% |
| prochlorperazine | 0 | 0.0% | 0 | 0.0% | 0 | 0.0% | 0 | 0.0% | 0 | 0.0% | 1 | 0.1% |
| progesterone | 3 | 0.1% | 0 | 0.0% | 1 | 0.1% | 1 | 0.2% | 0 | 0.0% | 7 | 0.5% |
| progestin | 1 | 0.0% | 0 | 0.0% | 0 | 0.0% | 0 | 0.0% | 0 | 0.0% | 1 | 0.1% |
| proparacaine | 0 | 0.0% | 0 | 0.0% | 0 | 0.0% | 0 | 0.0% | 0 | 0.0% | 1 | 0.1% |
| propofol | 0 | 0.0% | 0 | 0.0% | 0 | 0.0% | 0 | 0.0% | 1 | 0.1% | 0 | 0.0% |
| propoxyphene | 1 | 0.0% | 0 | 0.0% | 0 | 0.0% | 0 | 0.0% | 0 | 0.0% | 0 | 0.0% |
| propranolol | 1 | 0.0% | 0 | 0.0% | 1 | 0.1% | 0 | 0.0% | 0 | 0.0% | 2 | 0.1% |
| propylthiouracil | 1 | 0.0% | 0 | 0.0% | 1 | 0.1% | 0 | 0.0% | 1 | 0.1% | 1 | 0.1% |
| quetiapine | 0 | 0.0% | 0 | 0.0% | 1 | 0.1% | 0 | 0.0% | 1 | 0.1% | 0 | 0.0% |
| quetiapine fumarate | 0 | 0.0% | 3 | 0.8% | 0 | 0.0% | 1 | 0.2% | 0 | 0.0% | 4 | 0.3% |
| quinolone | 0 | 0.0% | 0 | 0.0% | 0 | 0.0% | 0 | 0.0% | 0 | 0.0% | 1 | 0.1% |
| rabeprazole | 1 | 0.0% | 1 | 0.3% | 0 | 0.0% | 2 | 0.3% | 0 | 0.0% | 3 | 0.2% |
| radiopharmaceutical | 1 | 0.0% | 0 | 0.0% | 0 | 0.0% | 0 | 0.0% | 0 | 0.0% | 0 | 0.0% |
| ramipril | 0 | 0.0% | 0 | 0.0% | 0 | 0.0% | 0 | 0.0% | 0 | 0.0% | 4 | 0.3% |
| ranitidine | 0 | 0.0% | 0 | 0.0% | 10 | 0.5% | 6 | 0.9% | 8 | 0.7% | 12 | 0.8% |
| reproductive control drug | 0 | 0.0% | 0 | 0.0% | 0 | 0.0% | 0 | 0.0% | 0 | 0.0% | 1 | 0.1% |
| risperidone | 0 | 0.0% | 0 | 0.0% | 0 | 0.0% | 0 | 0.0% | 1 | 0.1% | 0 | 0.0% |
| rizatriptan | 0 | 0.0% | 0 | 0.0% | 0 | 0.0% | 1 | 0.2% | 1 | 0.1% | 1 | 0.1% |
| salbutamol | 53 | 2.1% | 10 | 2.7% | 26 | 1.3% | 9 | 1.4% | 28 | 2.4% | 32 | 2.3% |
| salbutamol sulfate | 1 | 0.0% | 0 | 0.0% | 0 | 0.0% | 0 | 0.0% | 0 | 0.0% | 0 | 0.0% |
| scabicide | 0 | 0.0% | 2 | 0.5% | 0 | 0.0% | 0 | 0.0% | 0 | 0.0% | 0 | 0.0% |
| sertraline | 30 | 1.2% | 7 | 1.9% | 17 | 0.9% | 9 | 1.4% | 20 | 1.7% | 14 | 1.0% |
| spironolactone | 0 | 0.0% | 0 | 0.0% | 0 | 0.0% | 0 | 0.0% | 0 | 0.0% | 3 | 0.2% |
| steroid | 0 | 0.0% | 0 | 0.0% | 0 | 0.0% | 0 | 0.0% | 1 | 0.1% | 1 | 0.1% |
| sulfamethoxazole | 0 | 0.0% | 0 | 0.0% | 0 | 0.0% | 0 | 0.0% | 0 | 0.0% | 1 | 0.1% |
| sulfamethoxazole;trimethoprim | 6 | 0.2% | 1 | 0.3% | 1 | 0.1% | 0 | 0.0% | 2 | 0.2% | 2 | 0.1% |
| sulfasalazine | 1 | 0.0% | 0 | 0.0% | 1 | 0.1% | 1 | 0.2% | 0 | 0.0% | 3 | 0.2% |
| sulfonamide | 0 | 0.0% | 0 | 0.0% | 0 | 0.0% | 0 | 0.0% | 1 | 0.1% | 1 | 0.1% |
| sumatriptan | 0 | 0.0% | 0 | 0.0% | 0 | 0.0% | 1 | 0.2% | 1 | 0.1% | 0 | 0.0% |
| tacrolimus | 1 | 0.0% | 0 | 0.0% | 1 | 0.1% | 0 | 0.0% | 0 | 0.0% | 2 | 0.1% |
| tamsulosin | 0 | 0.0% | 0 | 0.0% | 0 | 0.0% | 0 | 0.0% | 1 | 0.1% | 0 | 0.0% |
| tazobactam | 1 | 0.0% | 0 | 0.0% | 0 | 0.0% | 0 | 0.0% | 0 | 0.0% | 0 | 0.0% |
| telmisartan | 0 | 0.0% | 0 | 0.0% | 0 | 0.0% | 1 | 0.2% | 0 | 0.0% | 0 | 0.0% |
| temazepam | 0 | 0.0% | 0 | 0.0% | 0 | 0.0% | 0 | 0.0% | 0 | 0.0% | 1 | 0.1% |
| terbinafine | 2 | 0.1% | 1 | 0.3% | 3 | 0.2% | 0 | 0.0% | 1 | 0.1% | 3 | 0.2% |
| terbinafine;terbinafine hydrochloride | 0 | 0.0% | 0 | 0.0% | 0 | 0.0% | 0 | 0.0% | 1 | 0.1% | 0 | 0.0% |
| terbutaline | 3 | 0.1% | 0 | 0.0% | 3 | 0.2% | 0 | 0.0% | 1 | 0.1% | 3 | 0.2% |
| terconazole | 1 | 0.0% | 0 | 0.0% | 0 | 0.0% | 0 | 0.0% | 0 | 0.0% | 1 | 0.1% |
| testosterone | 0 | 0.0% | 0 | 0.0% | 0 | 0.0% | 0 | 0.0% | 0 | 0.0% | 1 | 0.1% |
| thyroxine | 7 | 0.3% | 0 | 0.0% | 5 | 0.3% | 0 | 0.0% | 5 | 0.4% | 4 | 0.3% |
| tobramycin | 0 | 0.0% | 0 | 0.0% | 0 | 0.0% | 0 | 0.0% | 1 | 0.1% | 0 | 0.0% |
| topiramate | 0 | 0.0% | 1 | 0.3% | 0 | 0.0% | 1 | 0.2% | 0 | 0.0% | 2 | 0.1% |
| tramadol | 2 | 0.1% | 0 | 0.0% | 0 | 0.0% | 0 | 0.0% | 0 | 0.0% | 2 | 0.1% |
| tranexamic acid | 0 | 0.0% | 0 | 0.0% | 0 | 0.0% | 0 | 0.0% | 0 | 0.0% | 2 | 0.1% |
| tranylcypromine | 0 | 0.0% | 0 | 0.0% | 0 | 0.0% | 0 | 0.0% | 0 | 0.0% | 1 | 0.1% |
| trazodone | 2 | 0.1% | 0 | 0.0% | 0 | 0.0% | 0 | 0.0% | 1 | 0.1% | 1 | 0.1% |
| tretinoin | 1 | 0.0% | 0 | 0.0% | 1 | 0.1% | 0 | 0.0% | 0 | 0.0% | 1 | 0.1% |
| triamcinolone | 2 | 0.1% | 0 | 0.0% | 2 | 0.1% | 0 | 0.0% | 1 | 0.1% | 0 | 0.0% |
| trifluoperazine | 1 | 0.0% | 0 | 0.0% | 0 | 0.0% | 1 | 0.2% | 0 | 0.0% | 0 | 0.0% |
| trimethoprim | 1 | 0.0% | 0 | 0.0% | 0 | 0.0% | 0 | 0.0% | 0 | 0.0% | 1 | 0.1% |
| trimethoprim;sulfamethoxazole | 0 | 0.0% | 0 | 0.0% | 0 | 0.0% | 0 | 0.0% | 0 | 0.0% | 0 | 0.0% |
| ursodeoxycholic acid | 3 | 0.1% | 0 | 0.0% | 0 | 0.0% | 0 | 0.0% | 0 | 0.0% | 0 | 0.0% |
| vaccine | 6 | 0.2% | 0 | 0.0% | 3 | 0.2% | 0 | 0.0% | 3 | 0.3% | 0 | 0.0% |
| valacyclovir | 14 | 0.6% | 3 | 0.8% | 6 | 0.3% | 3 | 0.5% | 6 | 0.5% | 5 | 0.4% |
| valproic acid | 0 | 0.0% | 0 | 0.0% | 0 | 0.0% | 0 | 0.0% | 0 | 0.0% | 1 | 0.1% |
| varenicline | 0 | 0.0% | 0 | 0.0% | 0 | 0.0% | 0 | 0.0% | 0 | 0.0% | 3 | 0.2% |
| venlafaxine | 15 | 0.6% | 0 | 0.0% | 11 | 0.6% | 5 | 0.8% | 10 | 0.8% | 13 | 0.9% |
| vinblastine | 0 | 0.0% | 0 | 0.0% | 0 | 0.0% | 0 | 0.0% | 0 | 0.0% | 1 | 0.1% |
| warfarin | 2 | 0.1% | 0 | 0.0% | 2 | 0.1% | 1 | 0.2% | 0 | 0.0% | 1 | 0.1% |
| zinc sulfate | 1 | 0.0% | 0 | 0.0% | 0 | 0.0% | 0 | 0.0% | 0 | 0.0% | 0 | 0.0% |
| zolmitriptan | 0 | 0.0% | 1 | 0.3% | 0 | 0.0% | 0 | 0.0% | 0 | 0.0% | 2 | 0.1% |
| zopiclone | 3 | 0.1% | 1 | 0.3% | 1 | 0.1% | 3 | 0.5% | 1 | 0.1% | 16 | 1.1% |

**Additional file 3. List of all non-prescription medications and usage by breastfeeding and non-breastfeeding women at least once at 3, 6 and 12 months postpartum**

| **Non-prescription medications** | **N Women Breastfeeding at 3mos (n=2540)** | **%** | **N Women Non-breastfeeding at 3mos (n=366)** | **%** | **N Women Breastfeeding at 6mos (n=1984)** | **%** | **N Women Non-breastfeeding at 6mos (n=639)** | **%** | **N Women Breastfeeding at 12mos (n=1180)** | **%** | **N Women Non-breastfeeding at 12mos (n=1413)** | **%** |
| --- | --- | --- | --- | --- | --- | --- | --- | --- | --- | --- | --- | --- |
| 5-hydroxytryptophan | 0 | 0.0% | 0 | 0.0% | 1 | 0.1% | 0 | 0.0% | 0 | 0.0% | 2 | 0.1% |
| acacia fibre | 1 | 0.0% | 0 | 0.0% | 0 | 0.0% | 0 | 0.0% | 0 | 0.0% | 0 | 0.0% |
| acetaminophen | 264 | 10.4% | 41 | 11.2% | 229 | 11.5% | 65 | 10.2% | 137 | 11.6% | 164 | 11.6% |
| acetaminophen;acetylsalicylic acid;caffeine | 0 | 0.0% | 0 | 0.0% | 2 | 0.1% | 0 | 0.0% | 0 | 0.0% | 1 | 0.1% |
| acetaminophen;chlorpheniramine maleate;dextromethorphan hydrobromide;guaifenesin;pseudoephedrine hydrochloride | 1 | 0.0% | 0 | 0.0% | 0 | 0.0% | 0 | 0.0% | 0 | 0.0% | 0 | 0.0% |
| acetaminophen;chlorpheniramine;dextromethorphan;phenylephrine | 1 | 0.0% | 0 | 0.0% | 0 | 0.0% | 1 | 0.2% | 0 | 0.0% | 1 | 0.1% |
| acetaminophen;chlorpheniramine;phenylephrine | 1 | 0.0% | 0 | 0.0% | 0 | 0.0% | 0 | 0.0% | 0 | 0.0% | 0 | 0.0% |
| acetaminophen;diphenhydramine | 0 | 0.0% | 0 | 0.0% | 0 | 0.0% | 1 | 0.2% | 0 | 0.0% | 0 | 0.0% |
| acetaminophen;methocarbamol | 1 | 0.0% | 0 | 0.0% | 0 | 0.0% | 3 | 0.5% | 0 | 0.0% | 3 | 0.2% |
| acetaminophen;pheniramine;phenylephrine | 0 | 0.0% | 0 | 0.0% | 0 | 0.0% | 0 | 0.0% | 0 | 0.0% | 1 | 0.1% |
| acetaminophen;phenylephrine | 1 | 0.0% | 0 | 0.0% | 1 | 0.1% | 0 | 0.0% | 0 | 0.0% | 0 | 0.0% |
| acetaminophen;dextromethorpan hydrobromide;guaifenesin;pseudoephedrine hydrochloride | 0 | 0.0% | 0 | 0.0% | 1 | 0.1% | 0 | 0.0% | 0 | 0.0% | 0 | 0.0% |
| acetylsalicylic acid | 7 | 0.3% | 2 | 0.5% | 4 | 0.2% | 3 | 0.5% | 4 | 0.3% | 6 | 0.4% |
| acetylsalicylic acid;methocarbamol | 0 | 0.0% | 0 | 0.0% | 0 | 0.0% | 0 | 0.0% | 0 | 0.0% | 1 | 0.1% |
| adaptogenic herbs | 0 | 0.0% | 0 | 0.0% | 0 | 0.0% | 0 | 0.0% | 0 | 0.0% | 1 | 0.1% |
| adeeva nature's essential oils | 0 | 0.0% | 0 | 0.0% | 0 | 0.0% | 1 | 0.2% | 0 | 0.0% | 0 | 0.0% |
| adrenal px balance | 0 | 0.0% | 0 | 0.0% | 0 | 0.0% | 0 | 0.0% | 0 | 0.0% | 1 | 0.1% |
| adrenal sap | 1 | 0.0% | 0 | 0.0% | 0 | 0.0% | 0 | 0.0% | 0 | 0.0% | 0 | 0.0% |
| adrenergyn | 0 | 0.0% | 0 | 0.0% | 0 | 0.0% | 0 | 0.0% | 1 | 0.1% | 0 | 0.0% |
| ag-c | 1 | 0.0% | 0 | 0.0% | 0 | 0.0% | 0 | 0.0% | 0 | 0.0% | 0 | 0.0% |
| aged garlic extract | 0 | 0.0% | 0 | 0.0% | 1 | 0.1% | 0 | 0.0% | 0 | 0.0% | 0 | 0.0% |
| alfalfa | 2 | 0.1% | 0 | 0.0% | 1 | 0.1% | 0 | 0.0% | 1 | 0.1% | 0 | 0.0% |
| allergy essential oil blend | 0 | 0.0% | 0 | 0.0% | 1 | 0.1% | 0 | 0.0% | 0 | 0.0% | 0 | 0.0% |
| allimax garlic | 1 | 0.0% | 0 | 0.0% | 0 | 0.0% | 0 | 0.0% | 0 | 0.0% | 0 | 0.0% |
| aloe vera | 2 | 0.1% | 0 | 0.0% | 0 | 0.0% | 0 | 0.0% | 0 | 0.0% | 0 | 0.0% |
| aluminum acetate;benzethonium chloride | 0 | 0.0% | 0 | 0.0% | 0 | 0.0% | 1 | 0.2% | 0 | 0.0% | 0 | 0.0% |
| aluminum hydroxide;magnesium trisilicate | 7 | 0.3% | 1 | 0.3% | 2 | 0.1% | 1 | 0.2% | 2 | 0.2% | 2 | 0.1% |
| aluminum;magnesium | 2 | 0.1% | 1 | 0.3% | 1 | 0.1% | 0 | 0.0% | 0 | 0.0% | 0 | 0.0% |
| ammonium carbonate;camphor;menthol;potassium bicarbonate | 0 | 0.0% | 0 | 0.0% | 0 | 0.0% | 2 | 0.3% | 0 | 0.0% | 0 | 0.0% |
| acetaminophen;ammonium carbonate;camphor;diphenhydramine hydrochloride;guaifenesin;menthol;potassium bicarbonate | 0 | 0.0% | 0 | 0.0% | 1 | 0.1% | 0 | 0.0% | 0 | 0.0% | 0 | 0.0% |
| analgesic | 0 | 0.0% | 0 | 0.0% | 0 | 0.0% | 1 | 0.2% | 0 | 0.0% | 1 | 0.1% |
| anthelminthic drug unknown | 1 | 0.0% | 0 | 0.0% | 0 | 0.0% | 0 | 0.0% | 0 | 0.0% | 0 | 0.0% |
| anti-allergic agent unknown | 1 | 0.0% | 0 | 0.0% | 0 | 0.0% | 0 | 0.0% | 0 | 0.0% | 0 | 0.0% |
| anti-inflammatory drug unknown | 0 | 0.0% | 3 | 0.8% | 0 | 0.0% | 0 | 0.0% | 0 | 0.0% | 0 | 0.0% |
| antifungal drug | 0 | 0.0% | 0 | 0.0% | 0 | 0.0% | 0 | 0.0% | 0 | 0.0% | 2 | 0.1% |
| antifungal drug unknown | 2 | 0.1% | 0 | 0.0% | 0 | 0.0% | 0 | 0.0% | 0 | 0.0% | 0 | 0.0% |
| antioxidant | 3 | 0.1% | 0 | 0.0% | 1 | 0.1% | 0 | 0.0% | 0 | 0.0% | 2 | 0.1% |
| antioxidant vitamin | 0 | 0.0% | 0 | 0.0% | 1 | 0.1% | 0 | 0.0% | 0 | 0.0% | 0 | 0.0% |
| antipruritic drug unknown | 1 | 0.0% | 0 | 0.0% | 0 | 0.0% | 0 | 0.0% | 0 | 0.0% | 0 | 0.0% |
| antirheumatic drug | 0 | 0.0% | 0 | 0.0% | 0 | 0.0% | 0 | 0.0% | 2 | 0.2% | 0 | 0.0% |
| apothecare | 1 | 0.0% | 0 | 0.0% | 0 | 0.0% | 0 | 0.0% | 0 | 0.0% | 0 | 0.0% |
| apple cider vinegar | 0 | 0.0% | 0 | 0.0% | 1 | 0.1% | 0 | 0.0% | 0 | 0.0% | 0 | 0.0% |
| ar-encap | 0 | 0.0% | 0 | 0.0% | 0 | 0.0% | 0 | 0.0% | 0 | 0.0% | 1 | 0.1% |
| arnica montana | 1 | 0.0% | 0 | 0.0% | 0 | 0.0% | 0 | 0.0% | 0 | 0.0% | 0 | 0.0% |
| ascenta nutra sea | 1 | 0.0% | 0 | 0.0% | 0 | 0.0% | 0 | 0.0% | 0 | 0.0% | 0 | 0.0% |
| ascorbates | 1 | 0.0% | 0 | 0.0% | 0 | 0.0% | 0 | 0.0% | 0 | 0.0% | 0 | 0.0% |
| ascorbic acid | 0 | 0.0% | 0 | 0.0% | 1 | 0.1% | 0 | 0.0% | 2 | 0.2% | 0 | 0.0% |
| avena original liquid magnesium | 1 | 0.0% | 0 | 0.0% | 0 | 0.0% | 0 | 0.0% | 0 | 0.0% | 0 | 0.0% |
| avena originals full spectrum enzymes | 1 | 0.0% | 0 | 0.0% | 0 | 0.0% | 0 | 0.0% | 0 | 0.0% | 0 | 0.0% |
| b 100 complex | 0 | 0.0% | 0 | 0.0% | 1 | 0.1% | 0 | 0.0% | 0 | 0.0% | 1 | 0.1% |
| vitamin b12 | 27 | 1.1% | 1 | 0.3% | 20 | 1.0% | 6 | 0.9% | 8 | 0.7% | 6 | 0.4% |
| b complex | 0 | 0.0% | 0 | 0.0% | 1 | 0.1% | 0 | 0.0% | 1 | 0.1% | 0 | 0.0% |
| vitamin b50 | 4 | 0.2% | 0 | 0.0% | 2 | 0.1% | 1 | 0.2% | 3 | 0.3% | 1 | 0.1% |
| bach flower remedy | 0 | 0.0% | 0 | 0.0% | 1 | 0.1% | 0 | 0.0% | 0 | 0.0% | 0 | 0.0% |
| bach rescue remedy | 0 | 0.0% | 0 | 0.0% | 0 | 0.0% | 0 | 0.0% | 1 | 0.1% | 0 | 0.0% |
| bacitracin;polymyxin b sulfate | 3 | 0.1% | 0 | 0.0% | 2 | 0.1% | 0 | 0.0% | 3 | 0.3% | 0 | 0.0% |
| benefiber | 1 | 0.0% | 0 | 0.0% | 0 | 0.0% | 0 | 0.0% | 0 | 0.0% | 0 | 0.0% |
| benefibre | 8 | 0.3% | 0 | 0.0% | 2 | 0.1% | 0 | 0.0% | 0 | 0.0% | 0 | 0.0% |
| benzocaine | 0 | 0.0% | 0 | 0.0% | 0 | 0.0% | 0 | 0.0% | 1 | 0.1% | 0 | 0.0% |
| berberine | 0 | 0.0% | 0 | 0.0% | 1 | 0.1% | 0 | 0.0% | 0 | 0.0% | 0 | 0.0% |
| beta-carotene | 1 | 0.0% | 0 | 0.0% | 1 | 0.1% | 0 | 0.0% | 0 | 0.0% | 0 | 0.0% |
| bisacodyl | 0 | 0.0% | 0 | 0.0% | 0 | 0.0% | 0 | 0.0% | 0 | 0.0% | 1 | 0.1% |
| bismuth subsalicylate | 1 | 0.0% | 0 | 0.0% | 0 | 0.0% | 0 | 0.0% | 0 | 0.0% | 1 | 0.1% |
| bismuth subsalicylate;calcium carbonate | 1 | 0.0% | 0 | 0.0% | 1 | 0.1% | 1 | 0.2% | 0 | 0.0% | 0 | 0.0% |
| blessed thistle | 50 | 2.0% | 7 | 1.9% | 16 | 0.8% | 2 | 0.3% | 1 | 0.1% | 2 | 0.1% |
| blood builder iron supplement | 1 | 0.0% | 0 | 0.0% | 0 | 0.0% | 0 | 0.0% | 0 | 0.0% | 0 | 0.0% |
| boiron arnica montana | 1 | 0.0% | 0 | 0.0% | 0 | 0.0% | 0 | 0.0% | 0 | 0.0% | 0 | 0.0% |
| borage oil | 1 | 0.0% | 0 | 0.0% | 1 | 0.1% | 0 | 0.0% | 1 | 0.1% | 0 | 0.0% |
| brewer's yeast | 3 | 0.1% | 0 | 0.0% | 0 | 0.0% | 0 | 0.0% | 0 | 0.0% | 0 | 0.0% |
| butenafine | 1 | 0.0% | 0 | 0.0% | 0 | 0.0% | 0 | 0.0% | 0 | 0.0% | 0 | 0.0% |
| calcium | 213 | 8.4% | 8 | 2.2% | 103 | 5.2% | 8 | 1.3% | 62 | 5.3% | 37 | 2.6% |
| calcium carbonate | 36 | 1.4% | 6 | 1.6% | 31 | 1.6% | 7 | 1.1% | 16 | 1.4% | 14 | 1.0% |
| calcium carbonate;famotidine;magnesium hydroxide | 0 | 0.0% | 1 | 0.3% | 0 | 0.0% | 0 | 0.0% | 0 | 0.0% | 0 | 0.0% |
| calcium;magnesium | 8 | 0.3% | 2 | 0.5% | 2 | 0.1% | 0 | 0.0% | 1 | 0.1% | 0 | 0.0% |
| calcium citrate | 1 | 0.0% | 0 | 0.0% | 0 | 0.0% | 0 | 0.0% | 0 | 0.0% | 0 | 0.0% |
| calcium folate | 0 | 0.0% | 0 | 0.0% | 1 | 0.1% | 0 | 0.0% | 1 | 0.1% | 0 | 0.0% |
| calcium magnesium zinc | 1 | 0.0% | 0 | 0.0% | 0 | 0.0% | 0 | 0.0% | 0 | 0.0% | 0 | 0.0% |
| caltrate vitamin d and c | 1 | 0.0% | 0 | 0.0% | 0 | 0.0% | 0 | 0.0% | 0 | 0.0% | 0 | 0.0% |
| can albex 30x | 2 | 0.1% | 0 | 0.0% | 0 | 0.0% | 0 | 0.0% | 0 | 0.0% | 0 | 0.0% |
| cardiomiga 3 | 0 | 0.0% | 0 | 0.0% | 1 | 0.1% | 0 | 0.0% | 0 | 0.0% | 0 | 0.0% |
| centrum | 1 | 0.0% | 0 | 0.0% | 0 | 0.0% | 0 | 0.0% | 0 | 0.0% | 0 | 0.0% |
| cetirizine | 17 | 0.7% | 2 | 0.5% | 10 | 0.5% | 8 | 1.3% | 8 | 0.7% | 21 | 1.5% |
| chianutra | 1 | 0.0% | 0 | 0.0% | 0 | 0.0% | 0 | 0.0% | 0 | 0.0% | 0 | 0.0% |
| chinese herbs | 1 | 0.0% | 0 | 0.0% | 1 | 0.1% | 0 | 0.0% | 0 | 0.0% | 0 | 0.0% |
| chlorella | 1 | 0.0% | 0 | 0.0% | 0 | 0.0% | 0 | 0.0% | 0 | 0.0% | 0 | 0.0% |
| chlorella pyrenoidosa | 1 | 0.0% | 0 | 0.0% | 0 | 0.0% | 0 | 0.0% | 0 | 0.0% | 0 | 0.0% |
| chlorobutanol | 0 | 0.0% | 0 | 0.0% | 0 | 0.0% | 0 | 0.0% | 0 | 0.0% | 1 | 0.1% |
| chlorpheniramine | 1 | 0.0% | 0 | 0.0% | 1 | 0.1% | 0 | 0.0% | 0 | 0.0% | 2 | 0.1% |
| clotrimazole | 50 | 2.0% | 8 | 2.2% | 40 | 2.0% | 14 | 2.2% | 43 | 3.6% | 61 | 4.3% |
| co-q10 | 1 | 0.0% | 0 | 0.0% | 0 | 0.0% | 0 | 0.0% | 0 | 0.0% | 0 | 0.0% |
| cod liver oil | 9 | 0.4% | 1 | 0.3% | 2 | 0.1% | 0 | 0.0% | 1 | 0.1% | 4 | 0.3% |
| coenzyme b food complex | 0 | 0.0% | 1 | 0.3% | 0 | 0.0% | 0 | 0.0% | 0 | 0.0% | 0 | 0.0% |
| coenzyme q | 1 | 0.0% | 0 | 0.0% | 0 | 0.0% | 1 | 0.2% | 0 | 0.0% | 1 | 0.1% |
| coenzyme q10 | 1 | 0.0% | 0 | 0.0% | 2 | 0.1% | 1 | 0.2% | 0 | 0.0% | 2 | 0.1% |
| cold fx | 0 | 0.0% | 0 | 0.0% | 0 | 0.0% | 1 | 0.2% | 0 | 0.0% | 1 | 0.1% |
| collagen | 2 | 0.1% | 0 | 0.0% | 0 | 0.0% | 0 | 0.0% | 0 | 0.0% | 0 | 0.0% |
| complete b | 1 | 0.0% | 1 | 0.3% | 0 | 0.0% | 0 | 0.0% | 0 | 0.0% | 0 | 0.0% |
| coryzalia | 1 | 0.0% | 0 | 0.0% | 0 | 0.0% | 0 | 0.0% | 2 | 0.2% | 0 | 0.0% |
| cpink | 1 | 0.0% | 0 | 0.0% | 1 | 0.1% | 0 | 0.0% | 0 | 0.0% | 0 | 0.0% |
| cranberry extract | 2 | 0.1% | 0 | 0.0% | 3 | 0.2% | 0 | 0.0% | 0 | 0.0% | 2 | 0.1% |
| cranrich | 1 | 0.0% | 0 | 0.0% | 0 | 0.0% | 0 | 0.0% | 0 | 0.0% | 0 | 0.0% |
| cream | 0 | 0.0% | 0 | 0.0% | 1 | 0.1% | 0 | 0.0% | 0 | 0.0% | 0 | 0.0% |
| crystal violet | 11 | 0.4% | 0 | 0.0% | 1 | 0.1% | 0 | 0.0% | 0 | 0.0% | 0 | 0.0% |
| cyanocobalamin | 1 | 0.0% | 0 | 0.0% | 0 | 0.0% | 0 | 0.0% | 0 | 0.0% | 0 | 0.0% |
| d-mulsion 1000 | 2 | 0.1% | 0 | 0.0% | 0 | 0.0% | 0 | 0.0% | 0 | 0.0% | 0 | 0.0% |
| dairy digestive supplement | 1 | 0.0% | 0 | 0.0% | 0 | 0.0% | 0 | 0.0% | 0 | 0.0% | 0 | 0.0% |
| deep defense | 0 | 0.0% | 0 | 0.0% | 1 | 0.1% | 0 | 0.0% | 0 | 0.0% | 0 | 0.0% |
| desloratadine | 1 | 0.0% | 1 | 0.3% | 1 | 0.1% | 2 | 0.3% | 2 | 0.2% | 6 | 0.4% |
| dexbrompheniramine;pseudoephedrine | 0 | 0.0% | 0 | 0.0% | 1 | 0.1% | 0 | 0.0% | 0 | 0.0% | 1 | 0.1% |
| dexorange | 1 | 0.0% | 0 | 0.0% | 0 | 0.0% | 0 | 0.0% | 0 | 0.0% | 0 | 0.0% |
| dextromethorphan | 0 | 0.0% | 0 | 0.0% | 0 | 0.0% | 0 | 0.0% | 1 | 0.1% | 0 | 0.0% |
| dextromethorphan hydrobromide;diphenhydramine hydrochloride;guaifenesin;menthol;pseudoephedrine hydrochloride | 2 | 0.1% | 0 | 0.0% | 1 | 0.1% | 0 | 0.0% | 0 | 0.0% | 0 | 0.0% |
| acetaminophen; chlorpheniramine maleate;dextromethorphan hydrobromide;guaifenesin;pseudoephedrine hydrochloride | 0 | 0.0% | 0 | 0.0% | 0 | 0.0% | 1 | 0.2% | 0 | 0.0% | 0 | 0.0% |
| digest plus | 0 | 0.0% | 0 | 0.0% | 1 | 0.1% | 0 | 0.0% | 0 | 0.0% | 0 | 0.0% |
| digestive enzymes | 1 | 0.0% | 0 | 0.0% | 0 | 0.0% | 1 | 0.2% | 0 | 0.0% | 0 | 0.0% |
| digezyme | 0 | 0.0% | 0 | 0.0% | 0 | 0.0% | 0 | 0.0% | 1 | 0.1% | 0 | 0.0% |
| dimenhydrinate | 6 | 0.2% | 0 | 0.0% | 6 | 0.3% | 2 | 0.3% | 5 | 0.4% | 9 | 0.6% |
| diphenhydramine | 13 | 0.5% | 2 | 0.5% | 5 | 0.3% | 3 | 0.5% | 5 | 0.4% | 11 | 0.8% |
| docosahexaenoic acid | 2 | 0.1% | 0 | 0.0% | 0 | 0.0% | 0 | 0.0% | 0 | 0.0% | 0 | 0.0% |
| docosanol | 1 | 0.0% | 1 | 0.3% | 0 | 0.0% | 0 | 0.0% | 0 | 0.0% | 0 | 0.0% |
| docusate sodium | 59 | 2.3% | 7 | 1.9% | 8 | 0.4% | 3 | 0.5% | 1 | 0.1% | 1 | 0.1% |
| docusate calcium | 1 | 0.0% | 0 | 0.0% | 0 | 0.0% | 0 | 0.0% | 0 | 0.0% | 0 | 0.0% |
| doxylamine | 1 | 0.0% | 0 | 0.0% | 0 | 0.0% | 0 | 0.0% | 0 | 0.0% | 0 | 0.0% |
| dr reckeweg v-c 15 | 0 | 0.0% | 0 | 0.0% | 0 | 0.0% | 0 | 0.0% | 0 | 0.0% | 0 | 0.0% |
| drug cream | 0 | 0.0% | 0 | 0.0% | 1 | 0.1% | 0 | 0.0% | 2 | 0.2% | 0 | 0.0% |
| drug cream name unknown | 2 | 0.1% | 0 | 0.0% | 0 | 0.0% | 0 | 0.0% | 0 | 0.0% | 0 | 0.0% |
| earth mama organic milkmaid tea | 1 | 0.0% | 0 | 0.0% | 0 | 0.0% | 0 | 0.0% | 0 | 0.0% | 0 | 0.0% |
| echinacea | 4 | 0.2% | 0 | 0.0% | 1 | 0.1% | 0 | 0.0% | 1 | 0.1% | 0 | 0.0% |
| echinacea lozenges | 0 | 0.0% | 0 | 0.0% | 0 | 0.0% | 0 | 0.0% | 1 | 0.1% | 0 | 0.0% |
| echinacea zinc throat spray | 1 | 0.0% | 0 | 0.0% | 0 | 0.0% | 0 | 0.0% | 0 | 0.0% | 0 | 0.0% |
| efanatal | 0 | 0.0% | 0 | 0.0% | 1 | 0.1% | 0 | 0.0% | 0 | 0.0% | 0 | 0.0% |
| elderberry | 1 | 0.0% | 0 | 0.0% | 0 | 0.0% | 0 | 0.0% | 0 | 0.0% | 0 | 0.0% |
| elemental iron | 1 | 0.0% | 0 | 0.0% | 0 | 0.0% | 0 | 0.0% | 0 | 0.0% | 0 | 0.0% |
| elm powder | 1 | 0.0% | 0 | 0.0% | 0 | 0.0% | 0 | 0.0% | 0 | 0.0% | 0 | 0.0% |
| enfamil DHA | 0 | 0.0% | 0 | 0.0% | 2 | 0.1% | 0 | 0.0% | 0 | 0.0% | 0 | 0.0% |
| essential 3-6-9 | 1 | 0.0% | 0 | 0.0% | 0 | 0.0% | 0 | 0.0% | 0 | 0.0% | 0 | 0.0% |
| essential fatty acids | 0 | 0.0% | 0 | 0.0% | 0 | 0.0% | 0 | 0.0% | 1 | 0.1% | 0 | 0.0% |
| estriol | 0 | 0.0% | 0 | 0.0% | 1 | 0.1% | 0 | 0.0% | 0 | 0.0% | 0 | 0.0% |
| euro-fer | 0 | 0.0% | 0 | 0.0% | 1 | 0.1% | 0 | 0.0% | 1 | 0.1% | 0 | 0.0% |
| evening primrose | 0 | 0.0% | 0 | 0.0% | 0 | 0.0% | 0 | 0.0% | 1 | 0.1% | 0 | 0.0% |
| evening primrose oil | 7 | 0.3% | 0 | 0.0% | 3 | 0.2% | 0 | 0.0% | 0 | 0.0% | 1 | 0.1% |
| false unicorn | 1 | 0.0% | 0 | 0.0% | 0 | 0.0% | 0 | 0.0% | 0 | 0.0% | 0 | 0.0% |
| famotidine | 0 | 0.0% | 0 | 0.0% | 0 | 0.0% | 1 | 0.2% | 1 | 0.1% | 0 | 0.0% |
| fennel seed | 1 | 0.0% | 1 | 0.3% | 0 | 0.0% | 0 | 0.0% | 0 | 0.0% | 0 | 0.0% |
| fenugreek | 90 | 3.5% | 9 | 2.5% | 35 | 1.8% | 2 | 0.3% | 3 | 0.3% | 4 | 0.3% |
| fenugreek, dill, caraway, cumin, fennel, anise tea | 0 | 0.0% | 0 | 0.0% | 1 | 0.1% | 0 | 0.0% | 0 | 0.0% | 0 | 0.0% |
| feramax | 1 | 0.0% | 0 | 0.0% | 2 | 0.1% | 0 | 0.0% | 1 | 0.1% | 0 | 0.0% |
| feramax hematinic | 1 | 0.0% | 0 | 0.0% | 0 | 0.0% | 0 | 0.0% | 0 | 0.0% | 0 | 0.0% |
| feramax-150 | 0 | 0.0% | 0 | 0.0% | 0 | 0.0% | 0 | 0.0% | 1 | 0.1% | 0 | 0.0% |
| ferrasorb | 1 | 0.0% | 0 | 0.0% | 0 | 0.0% | 0 | 0.0% | 0 | 0.0% | 0 | 0.0% |
| ferrous ascorbate | 0 | 0.0% | 0 | 0.0% | 0 | 0.0% | 0 | 0.0% | 1 | 0.1% | 0 | 0.0% |
| ferrous fumarate | 26 | 1.0% | 4 | 1.1% | 7 | 0.4% | 1 | 0.2% | 2 | 0.2% | 5 | 0.4% |
| ferrous gluconate | 29 | 1.1% | 1 | 0.3% | 10 | 0.5% | 2 | 0.3% | 2 | 0.2% | 3 | 0.2% |
| ferrous sulfate | 7 | 0.3% | 0 | 0.0% | 5 | 0.3% | 0 | 0.0% | 4 | 0.3% | 2 | 0.1% |
| fexofenadine | 1 | 0.0% | 0 | 0.0% | 1 | 0.1% | 0 | 0.0% | 2 | 0.2% | 0 | 0.0% |
| fexofenadine;pseudoephedrine | 0 | 0.0% | 0 | 0.0% | 0 | 0.0% | 0 | 0.0% | 0 | 0.0% | 1 | 0.1% |
| fibre plus | 0 | 0.0% | 0 | 0.0% | 0 | 0.0% | 0 | 0.0% | 1 | 0.1% | 0 | 0.0% |
| finlandia lactation compound | 1 | 0.0% | 0 | 0.0% | 0 | 0.0% | 0 | 0.0% | 0 | 0.0% | 0 | 0.0% |
| finlandia lactation herbal | 1 | 0.0% | 0 | 0.0% | 0 | 0.0% | 0 | 0.0% | 0 | 0.0% | 0 | 0.0% |
| fish oil | 42 | 1.7% | 4 | 1.1% | 22 | 1.1% | 0 | 0.0% | 8 | 0.7% | 7 | 0.5% |
| fish oil dha | 0 | 0.0% | 0 | 0.0% | 1 | 0.1% | 0 | 0.0% | 0 | 0.0% | 1 | 0.1% |
| fish oil herbal magic | 0 | 0.0% | 0 | 0.0% | 0 | 0.0% | 0 | 0.0% | 1 | 0.1% | 0 | 0.0% |
| flax oil | 2 | 0.1% | 0 | 0.0% | 0 | 0.0% | 0 | 0.0% | 0 | 0.0% | 0 | 0.0% |
| flax omega 3 | 0 | 0.0% | 0 | 0.0% | 1 | 0.1% | 0 | 0.0% | 0 | 0.0% | 0 | 0.0% |
| flaxseed oil | 3 | 0.1% | 0 | 0.0% | 2 | 0.1% | 0 | 0.0% | 5 | 0.4% | 0 | 0.0% |
| floradix | 5 | 0.2% | 1 | 0.3% | 2 | 0.1% | 0 | 0.0% | 2 | 0.2% | 1 | 0.1% |
| floradix iron | 2 | 0.1% | 0 | 0.0% | 1 | 0.1% | 0 | 0.0% | 0 | 0.0% | 0 | 0.0% |
| fluconazole | 26 | 1.0% | 1 | 0.3% | 5 | 0.3% | 0 | 0.0% | 3 | 0.3% | 3 | 0.2% |
| fluticasone | 1 | 0.0% | 1 | 0.3% | 1 | 0.1% | 1 | 0.2% | 0 | 0.0% | 1 | 0.1% |
| folate | 1 | 0.0% | 1 | 0.3% | 1 | 0.1% | 0 | 0.0% | 0 | 0.0% | 0 | 0.0% |
| folic acid | 75 | 3.0% | 5 | 1.4% | 27 | 1.4% | 10 | 1.6% | 23 | 1.9% | 17 | 1.2% |
| folic herbal magic | 0 | 0.0% | 0 | 0.0% | 0 | 0.0% | 0 | 0.0% | 1 | 0.1% | 0 | 0.0% |
| forever living products | 0 | 0.0% | 1 | 0.3% | 0 | 0.0% | 0 | 0.0% | 0 | 0.0% | 0 | 0.0% |
| forever royal jelly | 0 | 0.0% | 0 | 0.0% | 0 | 0.0% | 1 | 0.2% | 0 | 0.0% | 0 | 0.0% |
| formula a7001 herbal magic | 0 | 0.0% | 0 | 0.0% | 0 | 0.0% | 0 | 0.0% | 1 | 0.1% | 0 | 0.0% |
| freeze-dried placenta | 1 | 0.0% | 0 | 0.0% | 0 | 0.0% | 0 | 0.0% | 0 | 0.0% | 0 | 0.0% |
| full spectrum digestive enzymes | 0 | 0.0% | 0 | 0.0% | 1 | 0.1% | 0 | 0.0% | 0 | 0.0% | 0 | 0.0% |
| fungisode | 1 | 0.0% | 0 | 0.0% | 0 | 0.0% | 0 | 0.0% | 0 | 0.0% | 0 | 0.0% |
| gaia mama's tea | 1 | 0.0% | 0 | 0.0% | 0 | 0.0% | 0 | 0.0% | 0 | 0.0% | 0 | 0.0% |
| garlic | 1 | 0.0% | 0 | 0.0% | 0 | 0.0% | 0 | 0.0% | 0 | 0.0% | 0 | 0.0% |
| garlic active principles | 0 | 0.0% | 0 | 0.0% | 0 | 0.0% | 0 | 0.0% | 1 | 0.1% | 0 | 0.0% |
| garlic extract | 1 | 0.0% | 0 | 0.0% | 0 | 0.0% | 0 | 0.0% | 0 | 0.0% | 0 | 0.0% |
| genestra calcium magnesium raspberry | 1 | 0.0% | 0 | 0.0% | 0 | 0.0% | 0 | 0.0% | 0 | 0.0% | 0 | 0.0% |
| gentle iron | 1 | 0.0% | 0 | 0.0% | 0 | 0.0% | 0 | 0.0% | 0 | 0.0% | 0 | 0.0% |
| genuine health greens+ instant smoothie | 1 | 0.0% | 0 | 0.0% | 0 | 0.0% | 0 | 0.0% | 0 | 0.0% | 0 | 0.0% |
| gin-chia | 0 | 0.0% | 0 | 0.0% | 0 | 0.0% | 1 | 0.2% | 0 | 0.0% | 0 | 0.0% |
| ginger | 1 | 0.0% | 0 | 0.0% | 0 | 0.0% | 0 | 0.0% | 0 | 0.0% | 0 | 0.0% |
| ginger gravol | 0 | 0.0% | 0 | 0.0% | 0 | 0.0% | 0 | 0.0% | 0 | 0.0% | 1 | 0.1% |
| ginger root | 0 | 0.0% | 0 | 0.0% | 1 | 0.1% | 0 | 0.0% | 0 | 0.0% | 0 | 0.0% |
| ginkgo | 0 | 0.0% | 0 | 0.0% | 0 | 0.0% | 0 | 0.0% | 1 | 0.1% | 0 | 0.0% |
| ginkgo biloba | 1 | 0.0% | 0 | 0.0% | 0 | 0.0% | 0 | 0.0% | 0 | 0.0% | 0 | 0.0% |
| glandular thyroid forte | 1 | 0.0% | 0 | 0.0% | 0 | 0.0% | 0 | 0.0% | 0 | 0.0% | 2 | 0.1% |
| glucoplex | 0 | 0.0% | 0 | 0.0% | 1 | 0.1% | 0 | 0.0% | 0 | 0.0% | 0 | 0.0% |
| glucosamine | 3 | 0.1% | 1 | 0.3% | 0 | 0.0% | 1 | 0.2% | 1 | 0.1% | 4 | 0.3% |
| glucosamine chondroitin | 0 | 0.0% | 0 | 0.0% | 1 | 0.1% | 0 | 0.0% | 0 | 0.0% | 1 | 0.1% |
| glucosamine study medicine | 0 | 0.0% | 0 | 0.0% | 0 | 0.0% | 0 | 0.0% | 0 | 0.0% | 1 | 0.1% |
| glucosamine sulfate | 0 | 0.0% | 0 | 0.0% | 1 | 0.1% | 0 | 0.0% | 1 | 0.1% | 1 | 0.1% |
| glutathione | 0 | 0.0% | 1 | 0.3% | 0 | 0.0% | 0 | 0.0% | 0 | 0.0% | 0 | 0.0% |
| glycopro b vitamin | 0 | 0.0% | 0 | 0.0% | 1 | 0.1% | 0 | 0.0% | 0 | 0.0% | 0 | 0.0% |
| goat's rue | 1 | 0.0% | 0 | 0.0% | 0 | 0.0% | 0 | 0.0% | 0 | 0.0% | 0 | 0.0% |
| gotu kola | 0 | 0.0% | 0 | 0.0% | 1 | 0.1% | 0 | 0.0% | 0 | 0.0% | 0 | 0.0% |
| grape seed extract | 2 | 0.1% | 0 | 0.0% | 1 | 0.1% | 0 | 0.0% | 0 | 0.0% | 0 | 0.0% |
| grapefruit complex liquid | 0 | 0.0% | 0 | 0.0% | 0 | 0.0% | 0 | 0.0% | 1 | 0.1% | 0 | 0.0% |
| grapefruit seed extract | 11 | 0.4% | 0 | 0.0% | 3 | 0.2% | 0 | 0.0% | 2 | 0.2% | 1 | 0.1% |
| greens phytonutrients probiotics and antioxidants | 1 | 0.0% | 0 | 0.0% | 0 | 0.0% | 0 | 0.0% | 0 | 0.0% | 0 | 0.0% |
| guaifenesin | 1 | 0.0% | 0 | 0.0% | 0 | 0.0% | 0 | 0.0% | 0 | 0.0% | 0 | 0.0% |
| hemagenics | 0 | 0.0% | 0 | 0.0% | 0 | 0.0% | 0 | 0.0% | 1 | 0.1% | 0 | 0.0% |
| hemoplex | 2 | 0.1% | 0 | 0.0% | 0 | 0.0% | 0 | 0.0% | 0 | 0.0% | 0 | 0.0% |
| hemp oil | 1 | 0.0% | 0 | 0.0% | 0 | 0.0% | 0 | 0.0% | 0 | 0.0% | 0 | 0.0% |
| henv3m re baby products | 1 | 0.0% | 0 | 0.0% | 0 | 0.0% | 0 | 0.0% | 0 | 0.0% | 0 | 0.0% |
| herbal bitters | 0 | 0.0% | 0 | 0.0% | 1 | 0.1% | 0 | 0.0% | 0 | 0.0% | 0 | 0.0% |
| herbal blend | 1 | 0.0% | 0 | 0.0% | 0 | 0.0% | 0 | 0.0% | 0 | 0.0% | 0 | 0.0% |
| herbal sunshine | 1 | 0.0% | 0 | 0.0% | 0 | 0.0% | 0 | 0.0% | 0 | 0.0% | 0 | 0.0% |
| herbal supplement | 0 | 0.0% | 0 | 0.0% | 1 | 0.1% | 0 | 0.0% | 0 | 0.0% | 0 | 0.0% |
| herbal tea | 1 | 0.0% | 0 | 0.0% | 0 | 0.0% | 0 | 0.0% | 0 | 0.0% | 0 | 0.0% |
| high potency calcium | 1 | 0.0% | 0 | 0.0% | 0 | 0.0% | 0 | 0.0% | 0 | 0.0% | 0 | 0.0% |
| hillestad summit stress | 0 | 0.0% | 0 | 0.0% | 0 | 0.0% | 0 | 0.0% | 0 | 0.0% | 1 | 0.1% |
| histamine antagonist | 2 | 0.1% | 0 | 0.0% | 0 | 0.0% | 0 | 0.0% | 0 | 0.0% | 0 | 0.0% |
| histaminum | 0 | 0.0% | 0 | 0.0% | 0 | 0.0% | 0 | 0.0% | 0 | 0.0% | 1 | 0.1% |
| homeopathy oscillo | 1 | 0.0% | 0 | 0.0% | 0 | 0.0% | 0 | 0.0% | 0 | 0.0% | 0 | 0.0% |
| hydroxycut | 0 | 0.0% | 0 | 0.0% | 0 | 0.0% | 0 | 0.0% | 0 | 0.0% | 1 | 0.1% |
| hyland's homeopathic | 0 | 0.0% | 0 | 0.0% | 1 | 0.1% | 0 | 0.0% | 0 | 0.0% | 0 | 0.0% |
| ibuprofen | 152 | 6.0% | 34 | 9.3% | 149 | 7.5% | 68 | 10.6% | 111 | 9.4% | 156 | 11.0% |
| ibuprofen;pseudoephedrine | 1 | 0.0% | 2 | 0.5% | 0 | 0.0% | 1 | 0.2% | 1 | 0.1% | 0 | 0.0% |
| ichol plus | 0 | 0.0% | 0 | 0.0% | 1 | 0.1% | 0 | 0.0% | 0 | 0.0% | 0 | 0.0% |
| immune 7 | 0 | 0.0% | 0 | 0.0% | 0 | 0.0% | 0 | 0.0% | 1 | 0.1% | 0 | 0.0% |
| immune 7 beyond immune support | 0 | 0.0% | 0 | 0.0% | 1 | 0.1% | 0 | 0.0% | 0 | 0.0% | 0 | 0.0% |
| inno-cal-mag complex | 1 | 0.0% | 0 | 0.0% | 0 | 0.0% | 0 | 0.0% | 0 | 0.0% | 0 | 0.0% |
| iodoral | 0 | 0.0% | 0 | 0.0% | 0 | 0.0% | 0 | 0.0% | 1 | 0.1% | 1 | 0.1% |
| iron | 90 | 3.5% | 9 | 2.5% | 17 | 0.9% | 5 | 0.8% | 6 | 0.5% | 13 | 0.9% |
| iron aspartate | 1 | 0.0% | 0 | 0.0% | 0 | 0.0% | 0 | 0.0% | 0 | 0.0% | 0 | 0.0% |
| iron citrate | 1 | 0.0% | 0 | 0.0% | 0 | 0.0% | 0 | 0.0% | 0 | 0.0% | 1 | 0.1% |
| iron complex | 1 | 0.0% | 1 | 0.3% | 0 | 0.0% | 0 | 0.0% | 0 | 0.0% | 0 | 0.0% |
| iron glycinate | 0 | 0.0% | 0 | 0.0% | 1 | 0.1% | 0 | 0.0% | 0 | 0.0% | 0 | 0.0% |
| iron pills | 0 | 0.0% | 0 | 0.0% | 0 | 0.0% | 0 | 0.0% | 0 | 0.0% | 1 | 0.1% |
| Iron sulfate | 1 | 0.0% | 0 | 0.0% | 0 | 0.0% | 0 | 0.0% | 0 | 0.0% | 0 | 0.0% |
| iron supplement | 0 | 0.0% | 0 | 0.0% | 0 | 0.0% | 0 | 0.0% | 1 | 0.1% | 0 | 0.0% |
| iron vital f | 0 | 0.0% | 0 | 0.0% | 0 | 0.0% | 0 | 0.0% | 1 | 0.1% | 0 | 0.0% |
| isys dry eye formula | 1 | 0.0% | 0 | 0.0% | 0 | 0.0% | 0 | 0.0% | 0 | 0.0% | 0 | 0.0% |
| jamieson b 100 | 1 | 0.0% | 0 | 0.0% | 0 | 0.0% | 0 | 0.0% | 0 | 0.0% | 1 | 0.1% |
| jamieson chewable vita-vim | 1 | 0.0% | 0 | 0.0% | 0 | 0.0% | 0 | 0.0% | 0 | 0.0% | 0 | 0.0% |
| jamieson wild salmon fish oil complex | 1 | 0.0% | 0 | 0.0% | 0 | 0.0% | 0 | 0.0% | 0 | 0.0% | 0 | 0.0% |
| joint care | 0 | 0.0% | 0 | 0.0% | 0 | 0.0% | 0 | 0.0% | 0 | 0.0% | 1 | 0.1% |
| juice orchard blend | 0 | 0.0% | 0 | 0.0% | 1 | 0.1% | 0 | 0.0% | 0 | 0.0% | 0 | 0.0% |
| juice plus | 0 | 0.0% | 0 | 0.0% | 0 | 0.0% | 0 | 0.0% | 0 | 0.0% | 1 | 0.1% |
| juice plus fruit juice chewable vitamin | 0 | 0.0% | 0 | 0.0% | 0 | 0.0% | 0 | 0.0% | 1 | 0.1% | 0 | 0.0% |
| juice plus garden blend | 1 | 0.0% | 0 | 0.0% | 0 | 0.0% | 0 | 0.0% | 0 | 0.0% | 0 | 0.0% |
| juice plus orchard blend | 1 | 0.0% | 0 | 0.0% | 0 | 0.0% | 0 | 0.0% | 0 | 0.0% | 0 | 0.0% |
| juice plus vegetable chewable vitamin | 0 | 0.0% | 0 | 0.0% | 0 | 0.0% | 0 | 0.0% | 1 | 0.1% | 0 | 0.0% |
| juice veggie blend | 0 | 0.0% | 0 | 0.0% | 1 | 0.1% | 0 | 0.0% | 0 | 0.0% | 0 | 0.0% |
| kai garmo | 1 | 0.0% | 0 | 0.0% | 0 | 0.0% | 0 | 0.0% | 0 | 0.0% | 0 | 0.0% |
| kelp | 2 | 0.1% | 0 | 0.0% | 0 | 0.0% | 0 | 0.0% | 0 | 0.0% | 0 | 0.0% |
| ketoconazole | 0 | 0.0% | 0 | 0.0% | 0 | 0.0% | 0 | 0.0% | 1 | 0.1% | 0 | 0.0% |
| klimakt-heel | 1 | 0.0% | 0 | 0.0% | 0 | 0.0% | 0 | 0.0% | 1 | 0.1% | 0 | 0.0% |
| krill oil | 0 | 0.0% | 0 | 0.0% | 2 | 0.1% | 0 | 0.0% | 0 | 0.0% | 0 | 0.0% |
| l lysine | 1 | 0.0% | 0 | 0.0% | 0 | 0.0% | 0 | 0.0% | 0 | 0.0% | 0 | 0.0% |
| L-5-methyltetrahydrofolate | 1 | 0.0% | 0 | 0.0% | 0 | 0.0% | 0 | 0.0% | 0 | 0.0% | 0 | 0.0% |
| lactaid | 1 | 0.0% | 0 | 0.0% | 0 | 0.0% | 0 | 0.0% | 0 | 0.0% | 0 | 0.0% |
| lactase enzyme | 0 | 0.0% | 0 | 0.0% | 0 | 0.0% | 1 | 0.2% | 0 | 0.0% | 0 | 0.0% |
| lactation compound name unknown | 1 | 0.0% | 0 | 0.0% | 0 | 0.0% | 0 | 0.0% | 0 | 0.0% | 0 | 0.0% |
| lactation herbal medication, name unknown | 0 | 0.0% | 0 | 0.0% | 1 | 0.1% | 0 | 0.0% | 0 | 0.0% | 0 | 0.0% |
| lactulose | 2 | 0.1% | 0 | 0.0% | 1 | 0.1% | 0 | 0.0% | 1 | 0.1% | 0 | 0.0% |
| Lanolin | 5 | 0.2% | 0 | 0.0% | 0 | 0.0% | 0 | 0.0% | 0 | 0.0% | 0 | 0.0% |
| laxative | 14 | 0.6% | 0 | 0.0% | 3 | 0.2% | 0 | 0.0% | 1 | 0.1% | 0 | 0.0% |
| leaves name unknown | 1 | 0.0% | 0 | 0.0% | 0 | 0.0% | 0 | 0.0% | 0 | 0.0% | 0 | 0.0% |
| lecithin | 10 | 0.4% | 0 | 0.0% | 6 | 0.3% | 0 | 0.0% | 4 | 0.3% | 0 | 0.0% |
| leptaden | 1 | 0.0% | 0 | 0.0% | 1 | 0.1% | 0 | 0.0% | 0 | 0.0% | 0 | 0.0% |
| levonorgestrel | 1 | 0.0% | 0 | 0.0% | 0 | 0.0% | 0 | 0.0% | 1 | 0.1% | 0 | 0.0% |
| lidocaine | 0 | 0.0% | 0 | 0.0% | 1 | 0.1% | 0 | 0.0% | 3 | 0.3% | 0 | 0.0% |
| lidocaine;sodium bicarbonate | 0 | 0.0% | 0 | 0.0% | 0 | 0.0% | 0 | 0.0% | 1 | 0.1% | 0 | 0.0% |
| life brand | 1 | 0.0% | 0 | 0.0% | 0 | 0.0% | 0 | 0.0% | 0 | 0.0% | 0 | 0.0% |
| lipoic acid | 0 | 0.0% | 0 | 0.0% | 0 | 0.0% | 0 | 0.0% | 0 | 0.0% | 1 | 0.1% |
| london naturals lozenge | 1 | 0.0% | 0 | 0.0% | 0 | 0.0% | 0 | 0.0% | 0 | 0.0% | 0 | 0.0% |
| loperamide | 1 | 0.0% | 0 | 0.0% | 0 | 0.0% | 0 | 0.0% | 1 | 0.1% | 1 | 0.1% |
| loratadine | 12 | 0.5% | 3 | 0.8% | 7 | 0.4% | 4 | 0.6% | 5 | 0.4% | 11 | 0.8% |
| lyphosot | 1 | 0.0% | 0 | 0.0% | 0 | 0.0% | 0 | 0.0% | 0 | 0.0% | 0 | 0.0% |
| maca | 1 | 0.0% | 0 | 0.0% | 0 | 0.0% | 0 | 0.0% | 0 | 0.0% | 0 | 0.0% |
| magnesium | 97 | 3.8% | 4 | 1.1% | 49 | 2.5% | 3 | 0.5% | 37 | 3.1% | 14 | 1.0% |
| malungai | 1 | 0.0% | 0 | 0.0% | 0 | 0.0% | 0 | 0.0% | 0 | 0.0% | 0 | 0.0% |
| malunggay | 0 | 0.0% | 0 | 0.0% | 1 | 0.1% | 0 | 0.0% | 0 | 0.0% | 0 | 0.0% |
| mannatech ambrotose | 0 | 0.0% | 0 | 0.0% | 0 | 0.0% | 0 | 0.0% | 1 | 0.1% | 0 | 0.0% |
| manuka honey | 1 | 0.0% | 0 | 0.0% | 0 | 0.0% | 0 | 0.0% | 0 | 0.0% | 0 | 0.0% |
| marshmallow herbal supplement | 0 | 0.0% | 0 | 0.0% | 1 | 0.1% | 0 | 0.0% | 0 | 0.0% | 0 | 0.0% |
| marshmallow root | 1 | 0.0% | 0 | 0.0% | 0 | 0.0% | 0 | 0.0% | 0 | 0.0% | 0 | 0.0% |
| materna | 0 | 0.0% | 0 | 0.0% | 7 | 0.4% | 0 | 0.0% | 1 | 0.1% | 3 | 0.2% |
| max krill oil | 0 | 0.0% | 0 | 0.0% | 0 | 0.0% | 0 | 0.0% | 0 | 0.0% | 1 | 0.1% |
| mega antioxidants | 1 | 0.0% | 0 | 0.0% | 1 | 0.1% | 0 | 0.0% | 0 | 0.0% | 0 | 0.0% |
| mega food whole food supplements | 1 | 0.0% | 0 | 0.0% | 0 | 0.0% | 0 | 0.0% | 0 | 0.0% | 0 | 0.0% |
| melatonin | 0 | 0.0% | 1 | 0.3% | 2 | 0.1% | 0 | 0.0% | 1 | 0.1% | 4 | 0.3% |
| menthol | 0 | 0.0% | 0 | 0.0% | 0 | 0.0% | 0 | 0.0% | 0 | 0.0% | 2 | 0.1% |
| mepivacaine hydrochloride | 0 | 0.0% | 0 | 0.0% | 1 | 0.1% | 0 | 0.0% | 0 | 0.0% | 0 | 0.0% |
| metagenics | 1 | 0.0% | 0 | 0.0% | 0 | 0.0% | 0 | 0.0% | 0 | 0.0% | 0 | 0.0% |
| metagenics wellness essential | 1 | 0.0% | 0 | 0.0% | 0 | 0.0% | 0 | 0.0% | 0 | 0.0% | 0 | 0.0% |
| metamucil | 9 | 0.4% | 0 | 0.0% | 4 | 0.2% | 0 | 0.0% | 2 | 0.2% | 4 | 0.3% |
| methocarbamol;acetaminophen | 0 | 0.0% | 0 | 0.0% | 0 | 0.0% | 0 | 0.0% | 1 | 0.1% | 0 | 0.0% |
| methocarbamol;ibuprofen | 0 | 0.0% | 0 | 0.0% | 0 | 0.0% | 0 | 0.0% | 0 | 0.0% | 2 | 0.1% |
| methyl mate b12 | 1 | 0.0% | 0 | 0.0% | 0 | 0.0% | 0 | 0.0% | 0 | 0.0% | 0 | 0.0% |
| methylsulfonylmethane | 0 | 0.0% | 0 | 0.0% | 1 | 0.1% | 0 | 0.0% | 0 | 0.0% | 0 | 0.0% |
| miconazole | 14 | 0.6% | 1 | 0.3% | 9 | 0.5% | 0 | 0.0% | 12 | 1.0% | 13 | 0.9% |
| milk thistle | 6 | 0.2% | 1 | 0.3% | 1 | 0.1% | 0 | 0.0% | 0 | 0.0% | 0 | 0.0% |
| milkmaid tea | 0 | 0.0% | 1 | 0.3% | 0 | 0.0% | 0 | 0.0% | 0 | 0.0% | 1 | 0.1% |
| mineral/vitamin supplement | 0 | 0.0% | 0 | 0.0% | 0 | 0.0% | 1 | 0.2% | 0 | 0.0% | 0 | 0.0% |
| minoxidil | 0 | 0.0% | 1 | 0.3% | 0 | 0.0% | 1 | 0.2% | 0 | 0.0% | 1 | 0.1% |
| mo milk mama | 0 | 0.0% | 0 | 0.0% | 1 | 0.1% | 0 | 0.0% | 0 | 0.0% | 0 | 0.0% |
| moducare | 1 | 0.0% | 0 | 0.0% | 0 | 0.0% | 0 | 0.0% | 0 | 0.0% | 0 | 0.0% |
| mometasone | 15 | 0.6% | 1 | 0.3% | 13 | 0.7% | 2 | 0.3% | 5 | 0.4% | 12 | 0.8% |
| more milk plus | 2 | 0.1% | 0 | 0.0% | 1 | 0.1% | 0 | 0.0% | 0 | 0.0% | 0 | 0.0% |
| moringa malunggam | 0 | 0.0% | 0 | 0.0% | 0 | 0.0% | 0 | 0.0% | 1 | 0.1% | 0 | 0.0% |
| mother's lactaflow | 1 | 0.0% | 0 | 0.0% | 0 | 0.0% | 0 | 0.0% | 0 | 0.0% | 0 | 0.0% |
| mother's milk herbal supplement | 0 | 0.0% | 0 | 0.0% | 1 | 0.1% | 0 | 0.0% | 0 | 0.0% | 0 | 0.0% |
| motherlove more milk plus | 1 | 0.0% | 0 | 0.0% | 1 | 0.1% | 0 | 0.0% | 0 | 0.0% | 0 | 0.0% |
| motherwort tea | 0 | 0.0% | 0 | 0.0% | 1 | 0.1% | 0 | 0.0% | 0 | 0.0% | 0 | 0.0% |
| mukosuta | 1 | 0.0% | 0 | 0.0% | 0 | 0.0% | 0 | 0.0% | 0 | 0.0% | 0 | 0.0% |
| multivitamin | 1228 | 48.3% | 93 | 25.4% | 563 | 28.4% | 61 | 9.5% | 306 | 25.9% | 215 | 15.2% |
| multimineral | 4 | 0.2% | 0 | 0.0% | 10 | 0.5% | 1 | 0.2% | 4 | 0.3% | 4 | 0.3% |
| multivitamin and mineral | 2 | 0.1% | 0 | 0.0% | 2 | 0.1% | 0 | 0.0% | 0 | 0.0% | 0 | 0.0% |
| multivitamin with dha | 1 | 0.0% | 0 | 0.0% | 0 | 0.0% | 0 | 0.0% | 0 | 0.0% | 0 | 0.0% |
| multivitamin with iron | 2 | 0.1% | 0 | 0.0% | 0 | 0.0% | 0 | 0.0% | 0 | 0.0% | 0 | 0.0% |
| mum ma milkstream | 0 | 0.0% | 0 | 0.0% | 1 | 0.1% | 0 | 0.0% | 0 | 0.0% | 0 | 0.0% |
| mupirocin | 21 | 0.8% | 2 | 0.5% | 2 | 0.1% | 1 | 0.2% | 2 | 0.2% | 1 | 0.1% |
| muscle relaxant | 0 | 0.0% | 0 | 0.0% | 1 | 0.1% | 0 | 0.0% | 0 | 0.0% | 1 | 0.1% |
| naproxen | 17 | 0.7% | 2 | 0.5% | 9 | 0.5% | 5 | 0.8% | 7 | 0.6% | 16 | 1.1% |
| nasal decongestant | 0 | 0.0% | 0 | 0.0% | 0 | 0.0% | 0 | 0.0% | 2 | 0.2% | 0 | 0.0% |
| nat sulph cell salts | 0 | 0.0% | 0 | 0.0% | 0 | 0.0% | 0 | 0.0% | 1 | 0.1% | 0 | 0.0% |
| natural antibiotic | 1 | 0.0% | 0 | 0.0% | 0 | 0.0% | 0 | 0.0% | 0 | 0.0% | 0 | 0.0% |
| nature's milk drops | 1 | 0.0% | 0 | 0.0% | 0 | 0.0% | 0 | 0.0% | 0 | 0.0% | 0 | 0.0% |
| nature's origin b complex | 0 | 0.0% | 0 | 0.0% | 0 | 0.0% | 0 | 0.0% | 0 | 0.0% | 1 | 0.1% |
| nature's sunshine | 1 | 0.0% | 0 | 0.0% | 1 | 0.1% | 0 | 0.0% | 1 | 0.1% | 0 | 0.0% |
| nervoheel | 0 | 0.0% | 0 | 0.0% | 0 | 0.0% | 0 | 0.0% | 1 | 0.1% | 0 | 0.0% |
| neurexan | 0 | 0.0% | 0 | 0.0% | 0 | 0.0% | 0 | 0.0% | 1 | 0.1% | 0 | 0.0% |
| new chapter bone strength | 0 | 0.0% | 0 | 0.0% | 1 | 0.1% | 0 | 0.0% | 0 | 0.0% | 0 | 0.0% |
| nfh gla sap borage oil | 0 | 0.0% | 0 | 0.0% | 1 | 0.1% | 0 | 0.0% | 0 | 0.0% | 0 | 0.0% |
| nfh l-lysine sap | 0 | 0.0% | 0 | 0.0% | 0 | 0.0% | 0 | 0.0% | 1 | 0.1% | 0 | 0.0% |
| nordic naturals | 1 | 0.0% | 0 | 0.0% | 0 | 0.0% | 0 | 0.0% | 0 | 0.0% | 0 | 0.0% |
| Novoferrogluc | 1 | 0.0% | 0 | 0.0% | 1 | 0.1% | 0 | 0.0% | 0 | 0.0% | 0 | 0.0% |
| nu life hemoplex | 1 | 0.0% | 0 | 0.0% | 0 | 0.0% | 0 | 0.0% | 0 | 0.0% | 0 | 0.0% |
| nursing mother's tea | 2 | 0.1% | 0 | 0.0% | 0 | 0.0% | 0 | 0.0% | 0 | 0.0% | 0 | 0.0% |
| nutrilite concentrated fruits and vegetables | 1 | 0.0% | 0 | 0.0% | 0 | 0.0% | 0 | 0.0% | 0 | 0.0% | 0 | 0.0% |
| oceans omega | 0 | 0.0% | 0 | 0.0% | 1 | 0.1% | 0 | 0.0% | 1 | 0.1% | 0 | 0.0% |
| oil of oregano | 4 | 0.2% | 1 | 0.3% | 1 | 0.1% | 0 | 0.0% | 0 | 0.0% | 0 | 0.0% |
| olive leaf | 1 | 0.0% | 0 | 0.0% | 0 | 0.0% | 0 | 0.0% | 0 | 0.0% | 0 | 0.0% |
| omega 3 | 204 | 8.0% | 8 | 2.2% | 86 | 4.3% | 9 | 1.4% | 64 | 5.4% | 30 | 2.1% |
| omega-3-6 | 1 | 0.0% | 0 | 0.0% | 0 | 0.0% | 1 | 0.2% | 0 | 0.0% | 1 | 0.1% |
| omega fish oil | 1 | 0.0% | 0 | 0.0% | 3 | 0.2% | 0 | 0.0% | 0 | 0.0% | 0 | 0.0% |
| omega 3 gummies | 1 | 0.0% | 0 | 0.0% | 1 | 0.1% | 0 | 0.0% | 0 | 0.0% | 0 | 0.0% |
| omega 3 prenatal epa/dha | 1 | 0.0% | 0 | 0.0% | 0 | 0.0% | 0 | 0.0% | 0 | 0.0% | 0 | 0.0% |
| omega 3 webber naturals | 1 | 0.0% | 0 | 0.0% | 0 | 0.0% | 0 | 0.0% | 0 | 0.0% | 0 | 0.0% |
| omega 3-5-6-7-8-9 | 0 | 0.0% | 0 | 0.0% | 1 | 0.1% | 0 | 0.0% | 0 | 0.0% | 0 | 0.0% |
| omega 3-6-9 | 30 | 1.2% | 1 | 0.3% | 23 | 1.2% | 4 | 0.6% | 12 | 1.0% | 8 | 0.6% |
| omega e-2 | 1 | 0.0% | 0 | 0.0% | 0 | 0.0% | 0 | 0.0% | 0 | 0.0% | 0 | 0.0% |
| omega factors | 1 | 0.0% | 0 | 0.0% | 0 | 0.0% | 0 | 0.0% | 0 | 0.0% | 0 | 0.0% |
| omega factors ultra prim | 1 | 0.0% | 0 | 0.0% | 0 | 0.0% | 0 | 0.0% | 0 | 0.0% | 0 | 0.0% |
| omega-3 with dha | 0 | 0.0% | 0 | 0.0% | 1 | 0.1% | 0 | 0.0% | 0 | 0.0% | 0 | 0.0% |
| omega-3-5-6-7-9 | 0 | 0.0% | 0 | 0.0% | 1 | 0.1% | 0 | 0.0% | 0 | 0.0% | 0 | 0.0% |
| omega-cla | 0 | 0.0% | 0 | 0.0% | 0 | 0.0% | 1 | 0.2% | 0 | 0.0% | 0 | 0.0% |
| organic raspberry leaf tea | 1 | 0.0% | 0 | 0.0% | 0 | 0.0% | 0 | 0.0% | 0 | 0.0% | 0 | 0.0% |
| organika fenugreek | 0 | 0.0% | 0 | 0.0% | 1 | 0.1% | 0 | 0.0% | 0 | 0.0% | 0 | 0.0% |
| organika professional line | 0 | 0.0% | 1 | 0.3% | 0 | 0.0% | 0 | 0.0% | 0 | 0.0% | 0 | 0.0% |
| ortho sleep | 0 | 0.0% | 0 | 0.0% | 0 | 0.0% | 0 | 0.0% | 1 | 0.1% | 0 | 0.0% |
| oscillococcinum | 1 | 0.0% | 0 | 0.0% | 0 | 0.0% | 0 | 0.0% | 0 | 0.0% | 0 | 0.0% |
| OTC | 1 | 0.0% | 0 | 0.0% | 0 | 0.0% | 0 | 0.0% | 0 | 0.0% | 0 | 0.0% |
| oxymetazoline | 1 | 0.0% | 0 | 0.0% | 0 | 0.0% | 0 | 0.0% | 0 | 0.0% | 1 | 0.1% |
| pacific salmon oil | 1 | 0.0% | 0 | 0.0% | 0 | 0.0% | 0 | 0.0% | 0 | 0.0% | 0 | 0.0% |
| palafer | 28 | 1.1% | 3 | 0.8% | 4 | 0.2% | 1 | 0.2% | 2 | 0.2% | 1 | 0.1% |
| palafer cf | 0 | 0.0% | 0 | 0.0% | 0 | 0.0% | 0 | 0.0% | 1 | 0.1% | 0 | 0.0% |
| palafer iron | 2 | 0.1% | 0 | 0.0% | 0 | 0.0% | 0 | 0.0% | 0 | 0.0% | 0 | 0.0% |
| pantothenic acid | 0 | 0.0% | 0 | 0.0% | 1 | 0.1% | 0 | 0.0% | 0 | 0.0% | 0 | 0.0% |
| papaya enzyme | 0 | 0.0% | 0 | 0.0% | 0 | 0.0% | 0 | 0.0% | 1 | 0.1% | 0 | 0.0% |
| pectin | 0 | 0.0% | 0 | 0.0% | 0 | 0.0% | 0 | 0.0% | 1 | 0.1% | 0 | 0.0% |
| phenylephrine | 2 | 0.1% | 0 | 0.0% | 0 | 0.0% | 0 | 0.0% | 0 | 0.0% | 0 | 0.0% |
| phenylephrine;pheniramine | 0 | 0.0% | 0 | 0.0% | 1 | 0.1% | 0 | 0.0% | 0 | 0.0% | 1 | 0.1% |
| phosphorus | 0 | 0.0% | 0 | 0.0% | 1 | 0.1% | 0 | 0.0% | 0 | 0.0% | 0 | 0.0% |
| phytomulti | 0 | 0.0% | 0 | 0.0% | 0 | 0.0% | 0 | 0.0% | 0 | 0.0% | 1 | 0.1% |
| phytosterols | 1 | 0.0% | 0 | 0.0% | 0 | 0.0% | 0 | 0.0% | 0 | 0.0% | 0 | 0.0% |
| placenta | 2 | 0.1% | 0 | 0.0% | 0 | 0.0% | 0 | 0.0% | 0 | 0.0% | 0 | 0.0% |
| placenta dehydrated | 2 | 0.1% | 0 | 0.0% | 0 | 0.0% | 0 | 0.0% | 0 | 0.0% | 0 | 0.0% |
| placenta encapsulation | 2 | 0.1% | 0 | 0.0% | 0 | 0.0% | 0 | 0.0% | 0 | 0.0% | 0 | 0.0% |
| placenta tablets | 1 | 0.0% | 0 | 0.0% | 0 | 0.0% | 0 | 0.0% | 0 | 0.0% | 0 | 0.0% |
| platinum easyiron | 0 | 0.0% | 0 | 0.0% | 1 | 0.1% | 0 | 0.0% | 0 | 0.0% | 0 | 0.0% |
| pleo muc | 0 | 0.0% | 0 | 0.0% | 0 | 0.0% | 0 | 0.0% | 0 | 0.0% | 1 | 0.1% |
| poliquin choline | 0 | 0.0% | 0 | 0.0% | 1 | 0.1% | 0 | 0.0% | 0 | 0.0% | 0 | 0.0% |
| polyethylene glycol | 3 | 0.1% | 0 | 0.0% | 2 | 0.1% | 0 | 0.0% | 1 | 0.1% | 1 | 0.1% |
| polysaccharide | 1 | 0.0% | 0 | 0.0% | 0 | 0.0% | 0 | 0.0% | 0 | 0.0% | 0 | 0.0% |
| potassium | 1 | 0.0% | 0 | 0.0% | 1 | 0.1% | 0 | 0.0% | 0 | 0.0% | 0 | 0.0% |
| potassium iodide | 1 | 0.0% | 0 | 0.0% | 0 | 0.0% | 0 | 0.0% | 0 | 0.0% | 0 | 0.0% |
| prairie doctor brand | 1 | 0.0% | 0 | 0.0% | 0 | 0.0% | 0 | 0.0% | 0 | 0.0% | 0 | 0.0% |
| prairie naturals hair force | 1 | 0.0% | 0 | 0.0% | 0 | 0.0% | 1 | 0.2% | 0 | 0.0% | 0 | 0.0% |
| prairie naturals ultra sil | 1 | 0.0% | 0 | 0.0% | 0 | 0.0% | 1 | 0.2% | 0 | 0.0% | 0 | 0.0% |
| pramocaine | 1 | 0.0% | 0 | 0.0% | 0 | 0.0% | 0 | 0.0% | 0 | 0.0% | 0 | 0.0% |
| pramoxine | 1 | 0.0% | 0 | 0.0% | 0 | 0.0% | 0 | 0.0% | 0 | 0.0% | 0 | 0.0% |
| pramoxine hydrochloride;zinc sulfate | 0 | 0.0% | 0 | 0.0% | 1 | 0.1% | 0 | 0.0% | 0 | 0.0% | 0 | 0.0% |
| pregvit | 2 | 0.1% | 1 | 0.3% | 0 | 0.0% | 0 | 0.0% | 0 | 0.0% | 0 | 0.0% |
| prenatal dha | 0 | 0.0% | 0 | 0.0% | 1 | 0.1% | 0 | 0.0% | 0 | 0.0% | 0 | 0.0% |
| prenatal multivitamins | 1 | 0.0% | 0 | 0.0% | 0 | 0.0% | 0 | 0.0% | 0 | 0.0% | 0 | 0.0% |
| prenatal vitamins | 7 | 0.3% | 0 | 0.0% | 1 | 0.1% | 0 | 0.0% | 0 | 0.0% | 0 | 0.0% |
| primrose oil | 0 | 0.0% | 0 | 0.0% | 1 | 0.1% | 0 | 0.0% | 0 | 0.0% | 1 | 0.1% |
| primrose oil with vitamin e | 1 | 0.0% | 0 | 0.0% | 0 | 0.0% | 0 | 0.0% | 0 | 0.0% | 0 | 0.0% |
| pro-gram cream | 0 | 0.0% | 0 | 0.0% | 1 | 0.1% | 0 | 0.0% | 0 | 0.0% | 0 | 0.0% |
| probiotic | 117 | 4.6% | 2 | 0.5% | 41 | 2.1% | 2 | 0.3% | 31 | 2.6% | 15 | 1.1% |
| proferrin | 1 | 0.0% | 0 | 0.0% | 0 | 0.0% | 0 | 0.0% | 1 | 0.1% | 0 | 0.0% |
| protein digestive aid | 2 | 0.1% | 0 | 0.0% | 0 | 0.0% | 0 | 0.0% | 0 | 0.0% | 0 | 0.0% |
| protein shake | 1 | 0.0% | 0 | 0.0% | 0 | 0.0% | 0 | 0.0% | 0 | 0.0% | 0 | 0.0% |
| acetaminophen;chlorpheniramine;pseudoephedrine | 0 | 0.0% | 0 | 0.0% | 0 | 0.0% | 0 | 0.0% | 2 | 0.2% | 1 | 0.1% |
| psyllium | 1 | 0.0% | 0 | 0.0% | 2 | 0.1% | 0 | 0.0% | 0 | 0.0% | 1 | 0.1% |
| pycnogenol | 1 | 0.0% | 0 | 0.0% | 1 | 0.1% | 0 | 0.0% | 0 | 0.0% | 0 | 0.0% |
| quarana | 1 | 0.0% | 0 | 0.0% | 0 | 0.0% | 0 | 0.0% | 0 | 0.0% | 0 | 0.0% |
| ranitidine | 28 | 1.1% | 5 | 1.4% | 0 | 0.0% | 0 | 0.0% | 0 | 0.0% | 0 | 0.0% |
| raspberry ketone | 0 | 0.0% | 0 | 0.0% | 0 | 0.0% | 0 | 0.0% | 0 | 0.0% | 2 | 0.1% |
| raspberry leaf | 1 | 0.0% | 0 | 0.0% | 1 | 0.1% | 0 | 0.0% | 0 | 0.0% | 0 | 0.0% |
| re9 collagen support | 0 | 0.0% | 0 | 0.0% | 0 | 0.0% | 0 | 0.0% | 0 | 0.0% | 1 | 0.1% |
| recovery freedom to move | 0 | 0.0% | 0 | 0.0% | 0 | 0.0% | 0 | 0.0% | 1 | 0.1% | 0 | 0.0% |
| red clover | 1 | 0.0% | 0 | 0.0% | 1 | 0.1% | 0 | 0.0% | 0 | 0.0% | 0 | 0.0% |
| red clover oil | 1 | 0.0% | 0 | 0.0% | 0 | 0.0% | 0 | 0.0% | 0 | 0.0% | 0 | 0.0% |
| red raspberry | 0 | 0.0% | 0 | 0.0% | 1 | 0.1% | 0 | 0.0% | 0 | 0.0% | 1 | 0.1% |
| red raspberry leaf, nettle, alfalfa tea | 0 | 0.0% | 0 | 0.0% | 1 | 0.1% | 0 | 0.0% | 0 | 0.0% | 0 | 0.0% |
| red reishi mushroom | 0 | 0.0% | 0 | 0.0% | 0 | 0.0% | 0 | 0.0% | 0 | 0.0% | 1 | 0.1% |
| rescue remedy | 0 | 0.0% | 0 | 0.0% | 1 | 0.1% | 0 | 0.0% | 0 | 0.0% | 0 | 0.0% |
| rhodiola | 1 | 0.0% | 0 | 0.0% | 2 | 0.1% | 0 | 0.0% | 1 | 0.1% | 1 | 0.1% |
| rosehips | 1 | 0.0% | 0 | 0.0% | 0 | 0.0% | 0 | 0.0% | 0 | 0.0% | 0 | 0.0% |
| saje natural remedies, aromatherapy, essential oils | 0 | 0.0% | 0 | 0.0% | 0 | 0.0% | 0 | 0.0% | 1 | 0.1% | 0 | 0.0% |
| salmon oil | 3 | 0.1% | 1 | 0.3% | 1 | 0.1% | 1 | 0.2% | 0 | 0.0% | 1 | 0.1% |
| sambu guard antiviral medication | 0 | 0.0% | 0 | 0.0% | 1 | 0.1% | 0 | 0.0% | 0 | 0.0% | 1 | 0.1% |
| scopolamine butylbromide | 2 | 0.1% | 0 | 0.0% | 2 | 0.1% | 0 | 0.0% | 1 | 0.1% | 1 | 0.1% |
| selenium | 1 | 0.0% | 0 | 0.0% | 0 | 0.0% | 0 | 0.0% | 1 | 0.1% | 0 | 0.0% |
| selenium sulfide | 1 | 0.0% | 0 | 0.0% | 0 | 0.0% | 0 | 0.0% | 0 | 0.0% | 0 | 0.0% |
| sennosides | 7 | 0.3% | 1 | 0.3% | 2 | 0.1% | 0 | 0.0% | 1 | 0.1% | 1 | 0.1% |
| shi quan da bu wan | 1 | 0.0% | 0 | 0.0% | 0 | 0.0% | 0 | 0.0% | 0 | 0.0% | 0 | 0.0% |
| shirafza | 1 | 0.0% | 0 | 0.0% | 0 | 0.0% | 0 | 0.0% | 0 | 0.0% | 0 | 0.0% |
| sho skin healing ointment | 0 | 0.0% | 0 | 0.0% | 0 | 0.0% | 0 | 0.0% | 1 | 0.1% | 0 | 0.0% |
| silica | 1 | 0.0% | 0 | 0.0% | 1 | 0.1% | 0 | 0.0% | 0 | 0.0% | 0 | 0.0% |
| simethicone | 0 | 0.0% | 0 | 0.0% | 1 | 0.1% | 0 | 0.0% | 1 | 0.1% | 0 | 0.0% |
| sodium chloride | 4 | 0.2% | 0 | 0.0% | 1 | 0.1% | 0 | 0.0% | 2 | 0.2% | 0 | 0.0% |
| sodium cromoglycate | 0 | 0.0% | 0 | 0.0% | 0 | 0.0% | 0 | 0.0% | 1 | 0.1% | 0 | 0.0% |
| soya lecithin | 1 | 0.0% | 0 | 0.0% | 0 | 0.0% | 0 | 0.0% | 0 | 0.0% | 0 | 0.0% |
| spectramin chelate | 0 | 0.0% | 0 | 0.0% | 0 | 0.0% | 0 | 0.0% | 1 | 0.1% | 0 | 0.0% |
| squid oil | 1 | 0.0% | 0 | 0.0% | 0 | 0.0% | 0 | 0.0% | 0 | 0.0% | 0 | 0.0% |
| st john's wort | 0 | 0.0% | 0 | 0.0% | 0 | 0.0% | 0 | 0.0% | 0 | 0.0% | 0 | 0.0% |
| staphysagria | 0 | 0.0% | 0 | 0.0% | 0 | 0.0% | 0 | 0.0% | 1 | 0.1% | 0 | 0.0% |
| sterculia | 0 | 0.0% | 0 | 0.0% | 0 | 0.0% | 0 | 0.0% | 1 | 0.1% | 0 | 0.0% |
| stodal | 1 | 0.0% | 0 | 0.0% | 0 | 0.0% | 0 | 0.0% | 1 | 0.1% | 0 | 0.0% |
| stress b formula with vitamin c | 0 | 0.0% | 0 | 0.0% | 0 | 0.0% | 0 | 0.0% | 1 | 0.1% | 0 | 0.0% |
| stress formula | 0 | 0.0% | 0 | 0.0% | 1 | 0.1% | 0 | 0.0% | 0 | 0.0% | 0 | 0.0% |
| stress vitamin | 1 | 0.0% | 0 | 0.0% | 0 | 0.0% | 0 | 0.0% | 0 | 0.0% | 0 | 0.0% |
| stress-ease b vitamin complex | 0 | 0.0% | 0 | 0.0% | 0 | 0.0% | 0 | 0.0% | 0 | 0.0% | 1 | 0.1% |
| stressease multimineral | 0 | 0.0% | 0 | 0.0% | 0 | 0.0% | 0 | 0.0% | 1 | 0.1% | 0 | 0.0% |
| stressease multivitamin | 0 | 0.0% | 0 | 0.0% | 0 | 0.0% | 0 | 0.0% | 1 | 0.1% | 0 | 0.0% |
| stresstabs | 0 | 0.0% | 0 | 0.0% | 1 | 0.1% | 1 | 0.2% | 0 | 0.0% | 0 | 0.0% |
| sulfacetamide sodium | 0 | 0.0% | 0 | 0.0% | 1 | 0.1% | 0 | 0.0% | 0 | 0.0% | 0 | 0.0% |
| sunrider | 1 | 0.0% | 0 | 0.0% | 0 | 0.0% | 0 | 0.0% | 0 | 0.0% | 0 | 0.0% |
| super b complex | 0 | 0.0% | 0 | 0.0% | 1 | 0.1% | 0 | 0.0% | 0 | 0.0% | 0 | 0.0% |
| swiss naturals cranberry | 1 | 0.0% | 0 | 0.0% | 0 | 0.0% | 1 | 0.2% | 0 | 0.0% | 0 | 0.0% |
| synergy dong quai | 1 | 0.0% | 0 | 0.0% | 0 | 0.0% | 0 | 0.0% | 0 | 0.0% | 0 | 0.0% |
| tetracaine;tetracaine hydrochloride | 0 | 0.0% | 0 | 0.0% | 0 | 0.0% | 0 | 0.0% | 0 | 0.0% | 1 | 0.1% |
| thorne ascorbic acid | 0 | 0.0% | 0 | 0.0% | 0 | 0.0% | 0 | 0.0% | 1 | 0.1% | 0 | 0.0% |
| thorne bio-gest | 1 | 0.0% | 0 | 0.0% | 0 | 0.0% | 0 | 0.0% | 0 | 0.0% | 0 | 0.0% |
| thyroid plus | 1 | 0.0% | 0 | 0.0% | 0 | 0.0% | 0 | 0.0% | 0 | 0.0% | 0 | 0.0% |
| tolnaftate | 0 | 0.0% | 0 | 0.0% | 1 | 0.1% | 0 | 0.0% | 1 | 0.1% | 0 | 0.0% |
| traumeel | 1 | 0.0% | 1 | 0.3% | 1 | 0.1% | 0 | 0.0% | 0 | 0.0% | 0 | 0.0% |
| tri iron folic | 1 | 0.0% | 0 | 0.0% | 0 | 0.0% | 0 | 0.0% | 0 | 0.0% | 0 | 0.0% |
| tri-boron plus | 0 | 0.0% | 0 | 0.0% | 1 | 0.1% | 0 | 0.0% | 0 | 0.0% | 0 | 0.0% |
| triamcinolone | 1 | 0.0% | 0 | 0.0% | 2 | 0.1% | 0 | 0.0% | 2 | 0.2% | 0 | 0.0% |
| triphlax | 0 | 0.0% | 0 | 0.0% | 0 | 0.0% | 0 | 0.0% | 1 | 0.1% | 0 | 0.0% |
| trophics | 1 | 0.0% | 0 | 0.0% | 0 | 0.0% | 0 | 0.0% | 0 | 0.0% | 0 | 0.0% |
| ubiquinol coqh | 0 | 0.0% | 0 | 0.0% | 1 | 0.1% | 0 | 0.0% | 0 | 0.0% | 0 | 0.0% |
| udo's choice dha | 1 | 0.0% | 0 | 0.0% | 0 | 0.0% | 0 | 0.0% | 0 | 0.0% | 0 | 0.0% |
| udo's essential oil | 0 | 0.0% | 0 | 0.0% | 1 | 0.1% | 0 | 0.0% | 0 | 0.0% | 0 | 0.0% |
| udo's omega | 1 | 0.0% | 0 | 0.0% | 0 | 0.0% | 0 | 0.0% | 0 | 0.0% | 0 | 0.0% |
| ultimate omega | 1 | 0.0% | 0 | 0.0% | 0 | 0.0% | 0 | 0.0% | 0 | 0.0% | 0 | 0.0% |
| ultra fibre | 0 | 0.0% | 0 | 0.0% | 1 | 0.1% | 0 | 0.0% | 0 | 0.0% | 0 | 0.0% |
| unda 1001 | 1 | 0.0% | 0 | 0.0% | 0 | 0.0% | 0 | 0.0% | 1 | 0.1% | 0 | 0.0% |
| unda 30 | 1 | 0.0% | 0 | 0.0% | 1 | 0.1% | 0 | 0.0% | 1 | 0.1% | 0 | 0.0% |
| unda homeopathic | 0 | 0.0% | 0 | 0.0% | 0 | 0.0% | 0 | 0.0% | 1 | 0.1% | 0 | 0.0% |
| unknown | 0 | 0.0% | 0 | 0.0% | 0 | 0.0% | 0 | 0.0% | 0 | 0.0% | 1 | 0.1% |
| usana | 1 | 0.0% | 0 | 0.0% | 0 | 0.0% | 0 | 0.0% | 0 | 0.0% | 0 | 0.0% |
| usana essentials vitamins and minerals | 0 | 0.0% | 0 | 0.0% | 1 | 0.1% | 0 | 0.0% | 0 | 0.0% | 0 | 0.0% |
| vega antioxidant omega oil blend | 0 | 0.0% | 0 | 0.0% | 0 | 0.0% | 0 | 0.0% | 1 | 0.1% | 0 | 0.0% |
| vega efa oil | 0 | 0.0% | 0 | 0.0% | 1 | 0.1% | 0 | 0.0% | 0 | 0.0% | 0 | 0.0% |
| vega one | 0 | 0.0% | 0 | 0.0% | 0 | 0.0% | 0 | 0.0% | 1 | 0.1% | 0 | 0.0% |
| vega whole meal replacement | 0 | 0.0% | 0 | 0.0% | 1 | 0.1% | 0 | 0.0% | 0 | 0.0% | 0 | 0.0% |
| vegetable mix supplement | 0 | 0.0% | 0 | 0.0% | 0 | 0.0% | 0 | 0.0% | 0 | 0.0% | 1 | 0.1% |
| vita aid | 1 | 0.0% | 0 | 0.0% | 0 | 0.0% | 0 | 0.0% | 0 | 0.0% | 0 | 0.0% |
| vitamin | 0 | 0.0% | 1 | 0.3% | 0 | 0.0% | 0 | 0.0% | 0 | 0.0% | 1 | 0.1% |
| vitamin d | 418 | 16.5% | 18 | 4.9% | 224 | 11.3% | 25 | 3.9% | 148 | 12.5% | 80 | 5.7% |
| vitamin a | 1 | 0.0% | 1 | 0.3% | 1 | 0.1% | 0 | 0.0% | 1 | 0.1% | 0 | 0.0% |
| vitamin b | 28 | 1.1% | 3 | 0.8% | 16 | 0.8% | 3 | 0.5% | 11 | 0.9% | 8 | 0.6% |
| vitamin b100 | 2 | 0.1% | 1 | 0.3% | 0 | 0.0% | 1 | 0.2% | 0 | 0.0% | 0 | 0.0% |
| vitamin b2 | 0 | 0.0% | 0 | 0.0% | 1 | 0.1% | 0 | 0.0% | 0 | 0.0% | 0 | 0.0% |
| vitamin b5 | 0 | 0.0% | 0 | 0.0% | 1 | 0.1% | 0 | 0.0% | 0 | 0.0% | 0 | 0.0% |
| vitamin b6 | 10 | 0.4% | 0 | 0.0% | 4 | 0.2% | 3 | 0.5% | 1 | 0.1% | 3 | 0.2% |
| vitamin b6 and b12 | 0 | 0.0% | 0 | 0.0% | 0 | 0.0% | 0 | 0.0% | 1 | 0.1% | 0 | 0.0% |
| vitamin c | 105 | 4.1% | 5 | 1.4% | 62 | 3.1% | 6 | 0.9% | 30 | 2.5% | 20 | 1.4% |
| Vitamin Code RAW Calcium | 0 | 0.0% | 0 | 0.0% | 1 | 0.1% | 0 | 0.0% | 0 | 0.0% | 0 | 0.0% |
| vitamin d drops | 1 | 0.0% | 1 | 0.3% | 0 | 0.0% | 0 | 0.0% | 0 | 0.0% | 0 | 0.0% |
| vitamin e | 14 | 0.6% | 0 | 0.0% | 3 | 0.2% | 2 | 0.3% | 1 | 0.1% | 3 | 0.2% |
| vitamin k | 1 | 0.0% | 0 | 0.0% | 1 | 0.1% | 0 | 0.0% | 0 | 0.0% | 0 | 0.0% |
| vitex | 1 | 0.0% | 0 | 0.0% | 0 | 0.0% | 0 | 0.0% | 0 | 0.0% | 0 | 0.0% |
| vogel echinacea | 1 | 0.0% | 0 | 0.0% | 0 | 0.0% | 0 | 0.0% | 0 | 0.0% | 0 | 0.0% |
| webber naturals | 2 | 0.1% | 0 | 0.0% | 0 | 0.0% | 0 | 0.0% | 1 | 0.1% | 0 | 0.0% |
| weleda nursing tea | 2 | 0.1% | 0 | 0.0% | 0 | 0.0% | 0 | 0.0% | 0 | 0.0% | 0 | 0.0% |
| wheat grass powder | 0 | 0.0% | 0 | 0.0% | 0 | 0.0% | 0 | 0.0% | 1 | 0.1% | 0 | 0.0% |
| wheatgrass and greens | 1 | 0.0% | 0 | 0.0% | 0 | 0.0% | 0 | 0.0% | 0 | 0.0% | 0 | 0.0% |
| wholemega | 1 | 0.0% | 0 | 0.0% | 0 | 0.0% | 0 | 0.0% | 0 | 0.0% | 0 | 0.0% |
| wild salmon oil | 1 | 0.0% | 0 | 0.0% | 0 | 0.0% | 0 | 0.0% | 0 | 0.0% | 0 | 0.0% |
| Witch Hazel-containing Anti-Hemorroidal Formulation | 1 | 0.0% | 0 | 0.0% | 0 | 0.0% | 0 | 0.0% | 0 | 0.0% | 0 | 0.0% |
| xylometazoline | 1 | 0.0% | 0 | 0.0% | 2 | 0.1% | 1 | 0.2% | 2 | 0.2% | 2 | 0.1% |
| yin chiao chien tu pien | 1 | 0.0% | 0 | 0.0% | 0 | 0.0% | 0 | 0.0% | 0 | 0.0% | 0 | 0.0% |
| zinc | 15 | 0.6% | 2 | 0.5% | 9 | 0.5% | 0 | 0.0% | 4 | 0.3% | 3 | 0.2% |
| zinc citrate | 1 | 0.0% | 0 | 0.0% | 0 | 0.0% | 0 | 0.0% | 0 | 0.0% | 0 | 0.0% |
| zinc lozenges with vitamin c | 0 | 0.0% | 0 | 0.0% | 0 | 0.0% | 0 | 0.0% | 1 | 0.1% | 0 | 0.0% |
| zinc picolinate | 1 | 0.0% | 0 | 0.0% | 0 | 0.0% | 0 | 0.0% | 0 | 0.0% | 0 | 0.0% |
| zinc sulfate | 3 | 0.1% | 0 | 0.0% | 1 | 0.1% | 0 | 0.0% | 0 | 0.0% | 0 | 0.0% |
